# A new vertebrate fauna from the Lower Cretaceous Holly Creek Formation of the Trinity Group, southwest Arkansas, USA

**DOI:** 10.7717/peerj.12242

**Published:** 2021-10-21

**Authors:** Celina A. Suarez, Joseph Frederickson, Richard L. Cifelli, Jeffrey G. Pittman, Randall L. Nydam, ReBecca K. Hunt-Foster, Kirsty Morgan

**Affiliations:** 1 Department of Geosciences, University of Arkansas at Fayetteville, Fayetteville, Arkansas, USA; 2 Weis Earth Science Museum, University of Wisconsin Oshkosh Fox Cities Campus, Menasha, WI, USA; 3 Department of Vertebrate Paleontology, Sam Noble Oklahoma Museum of Natural History, University of Oklahoma, Norman, OK, USA; 4 Ouachita Mountains Biological Station, Mena, Arkansas, USA; 5 Arizona College of Osteopathic Medicine, Midwestern University, Glendale, Arizona, USA; 6 Dinosaur National Monument, Jensen, UT, USA

**Keywords:** Trinity Group, Early Cretaceous, Dinsaur, Crocodile, Lissamphibian, Pycnodont fish, Turtles, Chondrichthyes, Holly Creek Formation, Paleobiogeography

## Abstract

We present a previously discovered but undescribed late Early Cretaceous vertebrate fauna from the Holly Creek Formation of the Trinity Group in Arkansas. The site from the ancient Gulf Coast is dominated by semi-aquatic forms and preserves a diverse aquatic, semi-aquatic, and terrestrial fauna. Fishes include fresh- to brackish-water chondrichthyans and a variety of actinopterygians, including semionotids, an amiid, and a new pycnodontiform, *Anomoeodus caddoi* sp. nov. Semi-aquatic taxa include lissamphibians, the solemydid turtle *Naomichelys*, a trionychid turtle, and coelognathosuchian crocodyliforms. Among terrestrial forms are several members of Dinosauria and one or more squamates, one of which, *Sciroseps pawhuskai* gen. et sp. nov., is described herein. Among Dinosauria, both large and small theropods (*Acrocanthosaurus*, *Deinonychus*, and *Richardoestesia*) and titanosauriform sauropods are represented; herein we also report the first occurrence of a nodosaurid ankylosaur from the Trinity Group. The fauna of the Holly Creek Formation is similar to other, widely scattered late Early Cretaceous assemblages across North America and suggests the presence of a low-diversity, broadly distributed continental ecosystem of the Early Cretaceous following the Late Jurassic faunal turnover. This low-diversity ecosystem contrasts sharply with the highly diverse ecosystem which emerged by the Cenomanian. The contrast underpins the importance of vicariance as an evolutionary driver brought on by Sevier tectonics and climatic changes, such as rising sea level and formation of the Western Interior Seaway, impacting the early Late Cretaceous ecosystem.

## Introduction

The Trinity Group of Texas, Oklahoma, and Arkansas preserves a wide array of vertebrate trace and body fossils. In Arkansas, however, few body fossils have been recovered from the Trinity Group; a notable exception being the theropod dinosaur *Arkansaurus fridayi* (Hunt & Quinn, 2018). Instead, vertebrates have hitherto mainly been represented by trace fossils, primarily extensive sauropod trackways and, more recently, extensive theropod trackways (Pittman & Bell, 2002; Platt et al., 2018) from the De Queen Formation. The tracks from the De Queen are like those of the Glen Rose Formation of the Trinity Group, and the fauna of the Arkansas Trinity Group is thought to be roughly equivalent to those of the Trinity Group in Texas and Oklahoma (*e.g*., Pittman & Bell, 2002), including faunas from the Antlers, Twin Mountains, Glen Rose, and Paluxy formations. Underlying the De Queen Formation is the Holly Creek Formation (Vanderpool, 1928), a grey organic-rich silty mudstone with abundant plant fragments, charcoal, and secondary pyrite formation that is likely equivalent to the much more fossiliferous Antlers Formation of Oklahoma and Texas, from which the holotype of *Acrocanthosaurus atokensis* was found (Stovall & Langston, 1950). In the 1980s, during the course of field investigations on the De Queen sauropod trackways (Pittman & Gillette, 1989), one of us (JGP) collected vertebrate remains and fossiliferous rock matrix from the upper part of the Holly Creek Formation, which cropped out in a drainage ditch excavated by the mining company (formerly Briar Mining Site, now CertainTeed Mining) in association with a reservoir to hold wastewater and mined residues. Herein we describe the fauna and material excavated and screen-washed from the recovered sediment at this location. We use here the site name “Briar Site” to associate these body fossils with the fossil footprints in the De Queen Formation reported by Pittman & Gillette (1989). The body fossils in the Holly Creek Formation were recovered from an extension of a natural tributary of Bluff Creek. Fossiliferous beds of both formations extend westward into the adjacent Briar Creek stream drainage, making the Briar Site extend across several square kilometers. Although screen-washing and associated techniques (Cifelli, Madsen & Larson, 1996) are aimed at recovery of small vertebrates, such as lissamphibians, lizards, and mammals, the wide array of fossils recovered from the Holly Creek Formation samples large elements of the fauna as well. The goal of this work is to describe the taxonomic variety at the Briar Site, compare them to other late Early Cretaceous faunas in North America, and, from this comparison, comment on biodiversity of the late Early Cretaceous of North America. Other Lower Cretaceous units of North America with notable vertebrate faunas include the Cedar Mountain and Cloverly formations, deposited within the foreland basin of the Sevier Fold and Thrust Belt. In contrast, the Arundel Clay of Maryland was deposited along the Atlantic coastal plain. Interestingly, the faunas preserved within these formations are very similar, with common occurrences of titanosauriform sauropods, large carnivores such as *Acrocanthosaurus*, aquatic turtles such as *Naomichelys*, polacanthid ankylosaurs, and triconodontid and spalacotheriid mammals. Thus, comparison of the Holly Creek fauna will provide a more complete picture of late Early Cretaceous biodiversity and the linkages between historically well-known western and eastern faunas. Additional work will be required for detailed systematic relationships of the specimens found at the Briar Site.

**Figure 1:**
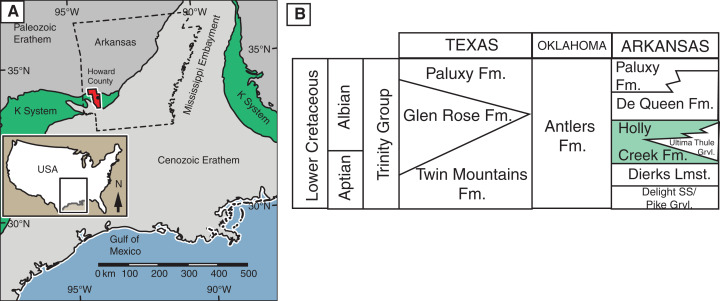
Location and stratigraphic position of Holly Creek Fauna. (A) Outcrop belt of the Cretaceous system relative to the Cenozoic and Paleozoic erathems in the Gulf Coast region; Howard County, Arkansas, in red. (B) Stratigraphic position of the Holly Creek Formation within the Arkansas Trinity Group, with its lateral equivalents of the Trinity Group in Texas and Oklahoma. Paleogeographic map modified from Platt et al. (2018). K-system = Cretaceous System.

**Geologic setting. **The Holly Creek Formation is part of the Lower Cretaceous Trinity Group. The Trinity Group, which crops out in the southwestern part of Arkansas and extends into Oklahoma and Texas, consists predominantly of an on-lap sequence of sandstone, claystone, and limestone, which rests unconformably on faulted and eroded Paleozoic beds (Vanderpool, 1928; Miser & Purdue, 1929; McFarland, 2004) on the west side of the Mississippi Embayment (Fig. 1A). In Arkansas, the Trinity Group consists of the Delight Sand/Pike Gravel, the Dierks Limestone, the Holly Creek Formation (which interfingers with the Ultima Thule Gravel), the De Queen Formation, and the Paluxy Formation (Vanderpool, 1928; Miser & Purdue, 1929; McFarland, 2004). In Oklahoma and adjacent parts of Texas, it consists almost entirely of the Antlers Formation, although the Holly Creek Formation is present in extreme southeast McCurtain County, Oklahoma. In central Texas, the Trinity Group consists of the Twin Mountains, Glen Rose, and Paluxy formations, in ascending order (Fig. 1B).

The upper part of the Holly Creek Formation, at least, is thought to be Albian in age. This is based on similarity of the oyster assemblage (*Ostrea franklini*) to that found in the overlying De Queen Formation (Miser & Purdue, 1929), suggesting an Albian age for the upper boundary of the Holly Creek Formation. Also, the presence of the foraminiferan *Orbitolina texana* in the Ferry Lake Anhydrite, a correlative unit to anhydrite beds of the lower De Queen Formation, and the presence of the ammonite *Douvilleiceras* sp. within the De Queen Formation units at the CertainTeed mine, support an Albian age (Loucks & Longman, 1982; Pittman, 1984, 1985). The occurrence, in the fossil-bearing beds of the Holly Creek Formation described here, of cheirolepidiacean conifer pollen cones (*Classostrobus arkansensis*) attached to *Pseudofrenelopsis parceramosa* (Watson, 1977), which ranges from Barremian to Cenomanian, is consistent with an Albian age for the Holly Creek Formation (Axsmith, Krings & Waselkov, 2004). Recent palynologic work on the correlative Glen Rose Formation of Texas suggests the abundance of *Classopollis* and *Exesipollenites* followed by reticulate monosulcate angiosperm pollen represents a late early Albian age (Tanrikulu, Doyle & Delusina, 2018). These samples were taken from above the *Corbula* beds that split the Glen Rose in Texas into an upper and lower member. Given that the overlying De Queen Formation is considered equivalent to the upper part of the Glen Rose, the Holly Creek Formation can be constrained to between early Albian and late Aptian.

Outcrops of the Holly Creek Formation are rare and typically restricted to creek beds or (as is the case for this material) mining operations. Our description of the strata of the Holly Creek Formation is based on observations by Pittman & Bell (2002) and on the matrix still attached to fossil material. It is composed of grey mudstones and fine-grained sandstones, with abundant organic material and charcoal as well as small, hard carbonate nodules. The abundance of organic material led to diagenetic development of abundant pyrite and marcasite nodules in and around bone. Also preserved within the matrix are whole charophyte gametangia.

## Materials and Methods

Larger macrovertebrate remains were excavated by co-author JGP and prepared either in the field or the lab at Lamar University. Undergraduate students at Lamar University, East Texas State University (now Texas A&M Commerce), and the University of Colorado at Denver helped with field collecting, processing, and sorting. Additional preparation was completed at the University of Arkansas and the Perot Museum of Nature and Science. Sediment was screen-washed and microvertebrates remains were picked at Lamar University and separated into vials based on taxonomic identification. Samples were then transferred to OMNH and sputter coated using a Denton Vacuum Desk II (gold/palladium) and imaged with an LEO 1450VP Scanning Electron Microscope by co-authors RLC and JAF. All macrovertebrate remains were imaged at the University of Arkansas and line drawings of identifiable and taxonomically significant remains were created in Adobe Photoshop and Illustrator by authors CAS and RLC. JPG used the following software for scanning and reconstructing a 3D model of one element: Adobe Photoshop for photo processing and masking; Agisoft Metashape for 3D reconstruction from photographs; Modo, by The Foundry Visionmongers, for mesh orientation and cleaning; NomadSculpt by Stephane Ginier for rotating and shifting mesh elements; Blender, by the Blender Foundation, for file format conversion; and Modo for video rendering. In total, 89 identifiable macrovertebrate specimens, several other unidentifiable fragments, and 1,347 individual microvertebrate samples (which includes multiple examples of the same taxa) form the basis for this report.

## Nomenclatural Acts

The electronic version of this article in Portable Document Format (PDF) will represent a published work according to the International Commission on Zoological Nomenclature (ICZN), and hence the new names contained in the electronic version are effectively published under that Code from the electronic edition alone. This published work and the nomenclatural acts it contains have been registered in ZooBank, the online registration system for the ICZN. The ZooBank LSIDs (Life Science Identifiers) can be resolved, and the associated information viewed through any standard web browser by appending the LSID to the prefix http://zoobank.org/. The LSID for this publication is: urn:lsid:zoobank.org:pub:E212457B-4FB0-48B9-BF64-01826BB2ADA4. The online version of this work is archived and available from the following digital repositories: PeerJ, PubMed Central and CLOCKSS.

## Systematic Paleontology

Vertebrate taxa from the Holly Creek Formation at the Briar Site are listed in Table 1. All material, with the exception of a coelognathosuchian skull that can be found at the Texas Memorial Museum at the University of Texas, is reposited at the UA. SEM images of microvertebrate fossils are reposited at both the UA and the OMNH. The following descriptive accounts and accompanying illustrations are intended to document the nature of the fossil material and our basis for identification.

**Table 1:** Faunal assemblage.

Chondrichthyes
Hybodontiformes
* Lonchidion anitae*
Lamniformes
gen. sp. indet.
Osteichthyes
Actinopterygii
Pycnodontiformes
* Anomoeodus caddoi* get. et sp. nov.
gen. sp. indet.
Semionotiformes
cf. *Lepidotes* sp.
Amiiformes
Amiidae indet.
Teleostei
gen. et sp. indet.
Lissamphibia
Anura
gen. et sp. indet.
Lepidosauria
Squamata
Scincomorpha Paramcellododae
* Sciroseps pawhuskai*, gen. et sp. nov.
gen. sp. indet.
Testudinata
Perichelydia
Solemydidae
* Naomichelys speciosa*
Cryptodira
Trionychoidea, indet.
Crocodylomorpha
Mesoeucrocodylia
Neosuchia
Coelognathosuchia indet.
* Paluxysuchus newmani*
cf. Bernissartiidae indet.
Dinosauria
Sauropoda
Titanisauriformes
cf. *Sauroposeidon* sp.
Theropoda
Carnosauria
* Acrocanthosaurus atokensis*
Coelurosauria
* Deinonychus antirrhopus*
cf. *Richardoestesia* sp.
Thyreophora
Ankylosauria
Nodosauridae, indet.
Mammalia
Eutriconodonta
Triconodontidae
Alticonodontinae, indet.
Trechnotheria
Spalacotheriidae
Spalacolestinae, indet.

**Note:**

Preliminary list of vertebrates from the Briar locality, Holly Creek Formation.

CHONDRICHTHYES Huxley, 1880

HYBDONTIFORMES Owen, 1846

LONCHIDIIDAE Herman, 1977

*Lonchidion anitae* Thurmond, 1971

**Referred material.** UA-2016-13-158, one complete tooth; UA-2016-13-159, one complete tooth; UA-2016-13-160, nine complete to partial teeth; UA-2016-13-161, 69 complete to partial teeth.

**Figure 2:**
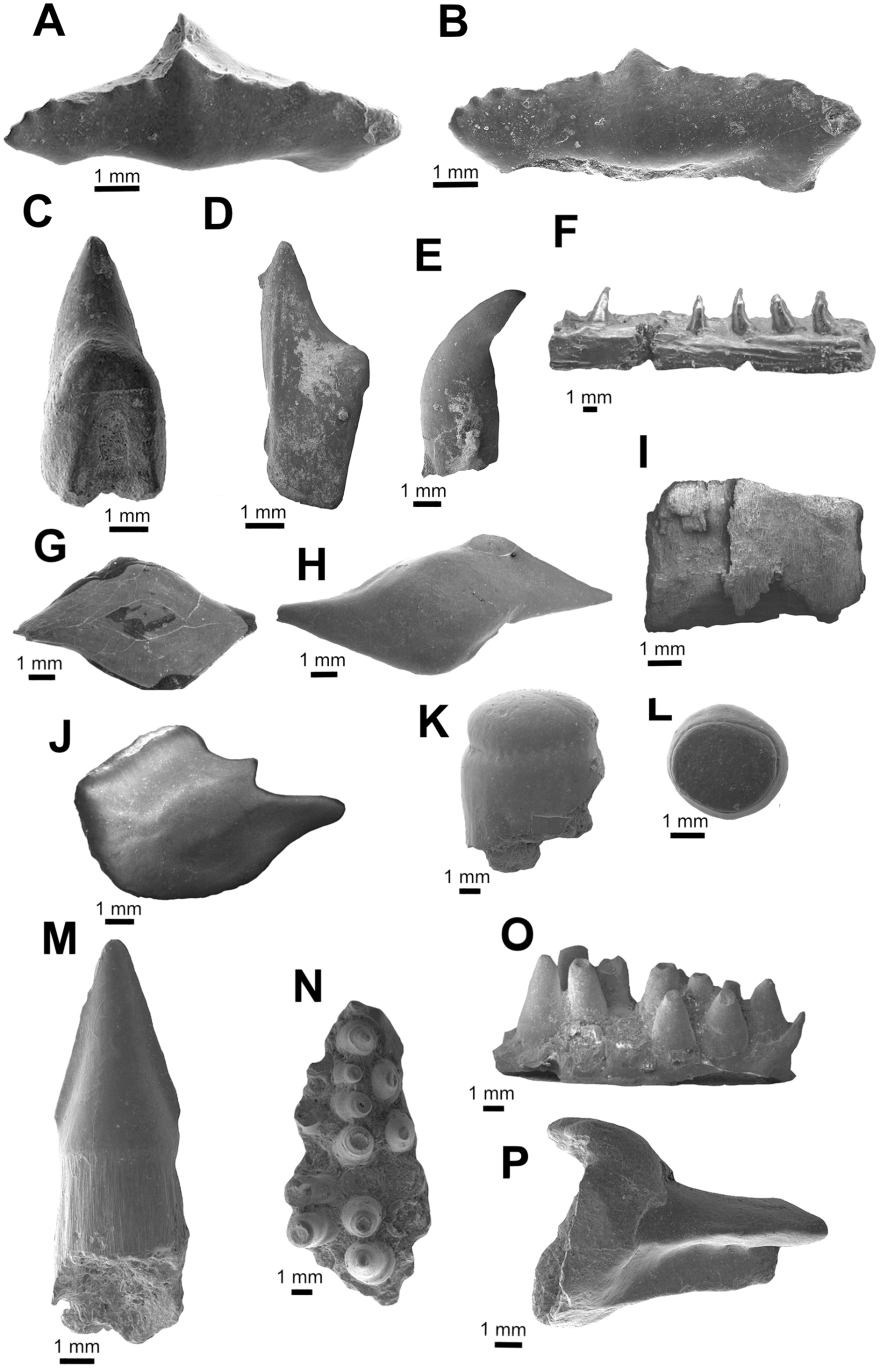
Chondrichthyes and Actinopterygii. (A–B) UA-2016-13-158, *Lonchidion anitae*, tooth in occlusal (A) and lingual (B) views. (C–D) UA-2016-13-157, indet. shark tooth in lingual (C) and lateral (D) views. (E–F) UA-2016-13-267 and UA-2016-13-269, Actinopterygii indet tooth (E) and jaw (F). (G–J) UA-2016-13-257a-c, unidentified ganoid scales. (K–L) UA-2016-13-263, cf. *Lepidotes* sp., teeth in lateral (K) and occlusal (L) views. (M) UA-2016-13-256, unidentified amiid tooth. (N–O) UA-2016-13-239, unidentified basibranchial teeth. (P) UA-2016-13-251, unidentified fish spine. Scale bars = one mm.

**Description and comments.** Several very small, triangular teeth (approximately 1–2 mm in mesiodistal width) with transversely expanded crowns are found at the Briar Site (Figs. 2A, 2B). The cutting surface of most teeth ranges from flat to weakly undulating, depending on the presence of accessory cusps. In most specimens, the lack of cusps is often associated with a clear wear facet, implying that absence of this feature is not taxonomically relevant. This material is here referred to *Lonchidion anitae*. Though most specimens lack the root, when present there is a noticeable constriction of the crown, as seen in other *Lonchidion* species (Sánchez-Hernández, Benton & Naish, 2007). These teeth are indistinguishable from those identified from the Antlers Formation of Oklahoma as “*Lissodus*” *anitae*, OMNH 32,309 and 61,457 from OMNH locality V706, Atoka County (Cifelli et al., 1997a), and specimens described from the Paluxy Formation of Texas (also referred to “*Lissodus*” *anitae*) (Welton & Farish, 1993). They are also similar to *Lonchidion* teeth described from the Cloverly Formation of Montana and Wyoming (Oreska, Carrano & Dzikiewicz, 2013).

LAMNIFORMES Garman, 1885.

Family, genus, and species indet.

**Referred material.** UA-2016-13-157, one incomplete tooth.

**Description and comments.** This single, small tooth bears a well-developed lingual protuberance on the root. Opposite of this, a deep and wide nutritive groove is present on the lingual face. The crown is low, straight, and relatively wide. It bears no cusplets or serrations and has only weakly defined striations near the interface with the root. The tooth is here identified as belonging to a yet undefined lamniform species (Figs. 2C, 2D). The tooth described here differs from the two species (both attributed to *Lamna*) identified by Thurmond (1971) from the Trinity Group of Texas in that both have accessory cusps and are larger. The reconstructed elongate root lobes and large root protuberance are most similar to *Paraisurus*, a genus that is relatively rare throughout the Albian to Cenomanian of Texas (Welton & Farish, 1993) and is not known from the Antlers of Oklahoma.

OSTEICHTHYES Huxley, 1880

ACTINOPTERYGII Klein, 1885

Family, genus, and species indet.

**Referred material.** UA-2016-13-251, UA-2016-13-257, UA-2016-13-266, UA-2016-13-269.

**Description and comments.** The isolated teeth and incomplete jaw listed under this heading are not currently identifiable beyond the fact that they belong to an actinopterygian (Figs. 2E, 2F). The teeth have basal fluting like those of amiiforms, differing in being capped by a small, rounded crown instead of the arrow-shaped apices commonly seen among amiiforms. The incomplete jaw element possesses five complete, recurved teeth arranged in an alternating line. The bone surface is highly irregular, with long grooves traversing the bone anteroposteriorly. Similar teeth are widely identified as both teleosts (Ouroumova, Shimada & Kirkland, 2016, fig. 3S) and non-teleosts (Nagrodski, Shimada & Schumacher, 2012, fig. 4J; Gallardo, Shimada & Schumacher, 2012, fig. 4E).

Ganoid fish scales are relatively common in the sample from the Holly Creek Formation; however, the lack of associated specimens and incomplete knowledge of North American Aptian/Albian fish squamation preclude more precise identification. The largest (morph I; Fig. 2J) have peg-and-socket articulations, an elongate rostral process, and a rounded caudal end. They are morphologically similar to those reported elsewhere from the Aptian/Albian of North America (scale type C of Frederickson, Lipka & Cifelli, 2018; morph D of Oreska, Carrano & Dzikiewicz, 2013). Morph II is the next largest; these scales are diamond-shaped and lack the peg-and-socket joint (Fig. 2G). Some of the largest specimens have hyper-elongate caudal and rostral processes and inflated median sides (Fig. 2H). Type III includes scales that are nearly square, with a peg on the median surface (Fig. 2I). There are also scales that are similar to the triangular-shaped and elongate scales of the dorsal series of some semionotids (Olsen & McCune, 1991). Multiple small vertebrae are also recognized as Actinopterygii indet. and are relatively common. These are all amphicoelous and often bear a centrally placed notochordal foramen in the centrum. Refined identification of these specimens may prove feasible following preparation and detailed study.

HOLOSTEI Müller, 1846.

SEMIONOTIFORMES Arambourg & Bertin, 1958.

SEMIONOTIDAE Woodward, 1890.

cf. *Lepidotes* Agassiz, 1832, sp. indet.

**Referred material.** UA-2016-13-221, 2016-13-222, 2016-13-263, 2016-13-267.

**Description and comments**. Semionotid teeth in the sample from the Holly Creek Formation are represented by two morphs: abundant, round oral teeth and rare (Figs. 2K–2L), tall pharyngeal teeth. The oral teeth are low crowned and bear a distinct enamel cap (Figs. 2K–2L). The pharyngeal teeth (2016-13-267) also possess this cap, but it is longer and more rod-like. These teeth are like those described as *Lepidotes* by Brinkman et al. (2013), (fig. 10.4A–E, G) and Oreska, Carrano & Dzikiewicz (2013), figs. 3C, D).

AMIIFORMES Hay, 1929.

AMIIDAE Bonaparte, 1837.

Gen. et sp. indet

**Referred material:** UA-2016-13-221; UA-2016-13-248, UA-2016-13-250, UA-2016-13-256, UA-2016-13-262.

**Figure 3:**
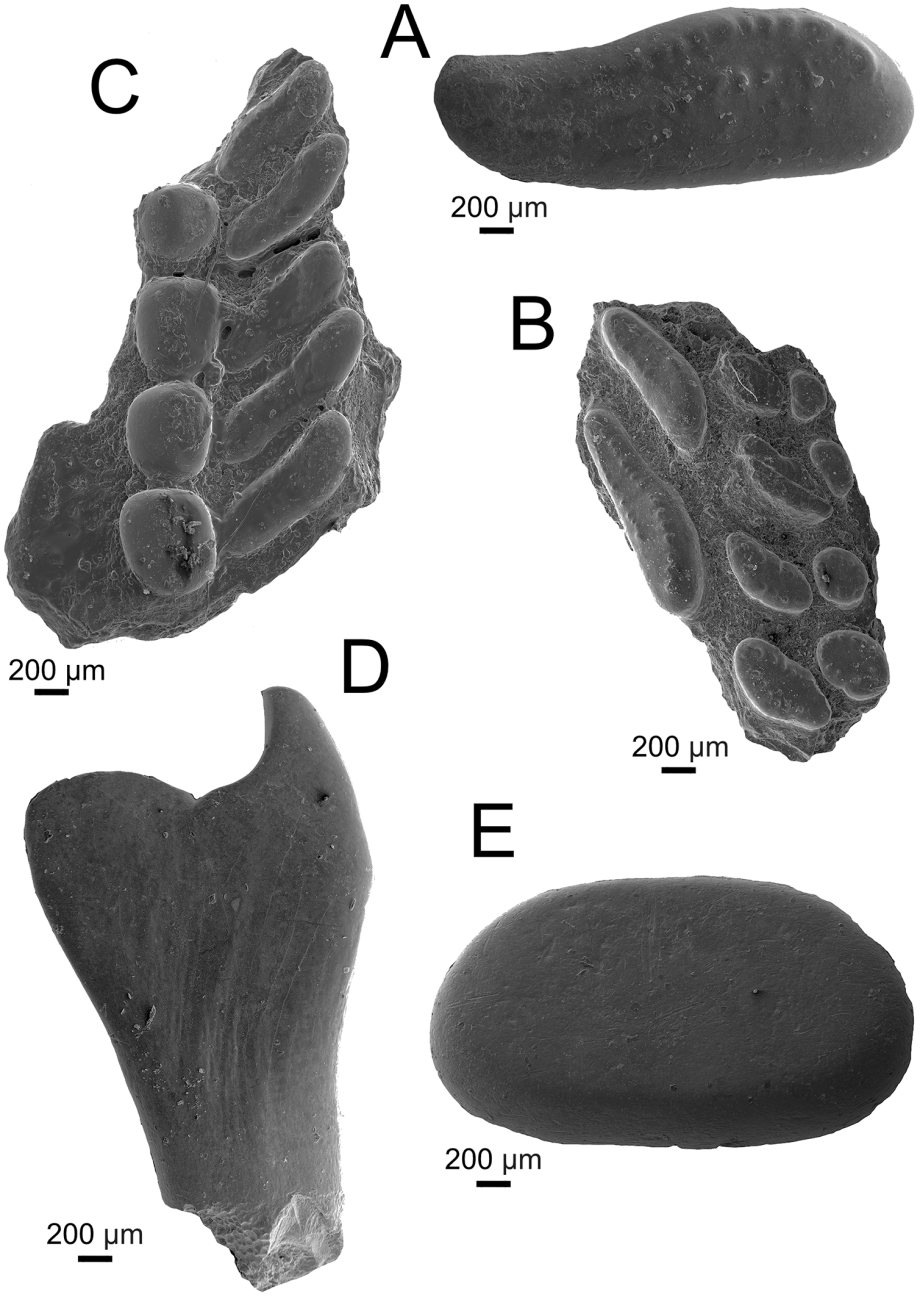
Pycnodontiformes fish teeth. (A–C) *Anomoeodus caddoi* sp. nov. (A) isolated tooth (UA-2016-13-291); (B) right prearticular plate (UA-2016-13-232, holotype); (C) left prearticular plate (UA-2016-13-220). (D-E) Pycnodontiformes, indet. (D) unidentified pharyngeal tooth (UA-2016-13-226); (E) isolated oral tooth (UA-2016-13-245). Scale bars = 200 μm.

**Description and comments**. Teeth with triangular, arrow-shaped cusps and weak basal fluting are found in screen-washed material. These teeth are here identified as amiiform (Fig. 2M). Teeth similar to these from the Trinity Group of Texas were originally assigned to “*Lepisosteus*” by Thurmond (1974). Bryant (1988) suggested the possibility that one such tooth, SMU-SMP 62252, might belong to the amiid *Melvius*; subsequent works have treated them as *Macrepistius* or an unidentified amiid (Bryant, 1988; Winkler, Murry & Jacobs, 1989, 1990; Barck, 1992). Similar teeth are also known from the contemporaneous Cloverly Formation (Oreska, Carrano & Dzikiewicz, 2013; fig. 3E) and Arundel Clay (Frederickson, Lipka & Cifelli, 2018).

TELEOSTEI Müller, 1845.

Family, genus, and species indet.

**Referred material:** UA-2016-13-228, UA**-**2016-13-239, UA-2016-13-259.

**Description and comments.** Tuberculate skull fragments (*e.g*., UA-2016-13-259) are fairly common in the sample from the Briar Site; these are similar to those figured by Becker & Chamberlain (2012), (fig. 5G) from the Cretaceous–Paleogene Clayton Limestone of Arkansas. In addition, closely-packed teeth that are rounded in cross section (UA-2016-13-228 and 2016-13-239, Figs. 2N, 2O) compare favorably to basibranchial teeth from the Late Cretaceous genus *Coriops* (Neuman & Brinkman, 2005; Brinkman et al., 2013, fig.9.6B, fig. 10.11B). Additionally, a few incomplete but elongate pieces with a cup-shaped end (UA 2016-13-251, Fig. 2P) are like teleost fish spines published elsewhere (Becker & Chamberlain, 2012, fig. 5A–C).

PYCNODONTIFORMES Berg, 1937.

Family incertae sedis.

***Anomoeodus caddoi* sp. nov.**

**Holotype**. UA-2016-13-232, prearticular plate bearing two main, four inner lateral, and four outer lateral teeth (Fig. 3B).

**Hypodigm**. The type, and UA-2016-13-220, prearticular plate preserving four inner lateral and five outermost lateral teeth (Fig. 3C); and UA-2016-13-291, a larger isolated main tooth (Fig. 3A).

**Type locality and horizon.** The holotype and all referred specimens were collected from the Briars Site, ca. 18 km NNW Nashville, Howard County, Arkansas (34.09°N, 93.88°W); uppermost Holly Creek Formation (Lower Cretaceous: Albian).

**Etymology.** The specific nomen refers to the Caddo Nation, Native American people who occupied a broad region of the lower Red River in Arkansas, Oklahoma, east Texas, and Louisiana.

**Diagnosis**. Small pycnodont with prearticular teeth generally like those of other *Anomoeodus* species. Main prearticular teeth elongate and reniform, distinct in being more compressed, elongate, S-shaped, and interspaced than in other *Anomoeodus* taxa. Lateral prearticular teeth oval to reniform and partially intercalated. Crimped-wall and coronal furlough very weakly-developed on all teeth. Second lateral row on prearticular has an anteromedial-tip that points weakly forward and caudo-lateral tip that points slightly backward.

**Description and comments.** The teeth in these specimens are small (less than two mm in length) and bear weak apical pits surrounded by crenulations (Fig. 3A). The crenulated crowns are similar to specimens referred to *Texasensis* Özdikmen, 2009 (formerly *Callodus* Thurmond, 1974), *Palaeobalistum* de Blainville, 1818, and *Anomoeodus* Forir, 1887, all of which occur in the Aptian–Albian of North America (Thurmond, 1974). Kriwet (2005), however, considers cusp ornamentation less taxonomically important than other characters, given its propensity to be lost in worn teeth. The holotype, a partial right anterior prearticular (Figs. 3B, 3C) preserves three rows of teeth, with an elongate main row and two additional lateral rows of reniform and rounded teeth respectively. The main prearticular teeth are elongate and obliquely arranged, bearing a weak anterior point; all of which are diagnostic of the genus *Anomoeodus* (Kriwet, 1999). Though known from over 25 species, most are defined entirely based on dental characteristics from incomplete material (Kriwet, 2002; Poyato-Ariza & Wenz, 2002; Shimada & Everhart, 2009). The Holly Creek material, though imperfect, is comparable to other known dentitions and most similar to *A. nursalli* from the Early Cretaceous (Barremian) of Spain (Kriwet, 1999). Prearticulars from this species are small and bear two medial, one main, and at least three lateral rows of teeth. UA-2016-13-232 is broken medially, anteriorly, and posteriorly, hindering identification of a precise count of tooth row number for the species. It lacks the innermost lateral row of *A. nursalli* which is similar in appearance to the main row but with the teeth arranged parallel to the oral border. This row disappears anteriorly in *A. nursalli*, making it impossible to determine if this row was truly missing in *A. caddoi*. The partial left prearticular described by Thurmond (1974) and likely belonging to *A. caddoi* or a closely related species, possesses this extra lateral row. The main teeth in *A. nursalli* have a well-defined crimped edge which is absent or only weakly present in *A. caddoi*. Larger and isolated main row teeth (UA-2016-13-291, Fig. 3A) are readily differentiated from the rest of the material based on the overall shape and crenulated occlusal surface. This tooth has a length/width ratio of 2.68, comparable to other species of *Anomoeodus* (Kriwet, 2002: table 2). The other prearticular from *A. caddoi* is a partial left (UA-2016-13-220; Fig. 3C) that preserves two incomplete lateral tooth rows: a large row bearing four large, rounded teeth: and another bearing five elongate, compressed teeth. We interpret this as preserving the two lateral-most rows located in the caudal portion of the jaw. These lateral tooth rows are consistent in shape between the two plates, where the outermost rows are spherical and the more inner row are weakly S-shaped, with a forward-facing anterior end and caudal-facing posterior end. The occlusal surfaces of the two preserved lateral rows meet at a relatively low, flat angle. Generally, the teeth are widely spaced and narrower than those of other *Anomoeodus* species. *Anomoeodus barberi* Hussakof, 1947 is another species known from the Cretaceous of Arkansas. This species, from the Upper Cretaceous Marlbrook Marl, has teeth that are unornamented and that are more ovate in the main row and more spherical in the inner-lateral row.

Genus and species indet.

**Referred material**. UA-2016-13-226, UA-2016-13-227, UA-2016-13-245, UA-2016-13-290.

**Description and comments**. Multiple pharyngeal teeth are identified as belonging to Pycnodontiformes indet. These teeth are relatively large (up to 3.1 mm in height), recurved, and may have an extremely long ventral blade (Fig. 3D). An additional oral tooth, larger (2.44 mm in length) than those of *Anomoeodus caddoi*, is also known (Fig. 3E). This tooth is highly rounded and lacks cusplets on the crown. As noted by Thurmond (1974), a complete reevaluation of the Early Cretaceous pycnodonts of Texas is needed; indeed, a comprehensive work focused largely on tooth morphology would greatly add to our knowledge of this group (Kriwet, 2005).

LISSAMPHIBIA Haeckel, 1866

**Description and comment.** Lissamphibian fossils from the Briar Site are highly fragmented, severely limiting taxonomic resolution. To date we have not positively identified specific elements other than the two anuran elements described below, but the collection includes squamous bone fragments bearing polygonal pits, which is consistent with ornamentation seen on *Albanerpeton* frontals (*e.g*., Gardner, 2000, 2001). At present we formally recognize only Anura among these fossils. *Albanerpeton* Estes & Hoffstetter, 1976, a commonly encountered faunal element of late Early and Late Cretaceous terrestrial assemblages of North America (Gardner & DeMar, 2013), is also likely present in the Holly Creek Formation.

ANURA Fischer von Waldheim, 1813

Family, genus, and species indet.

**Referred material.** UA-2016-13-155, partial tibia-fibula; UA-2016-13-145, partial maxilla.

**Figure 4:**
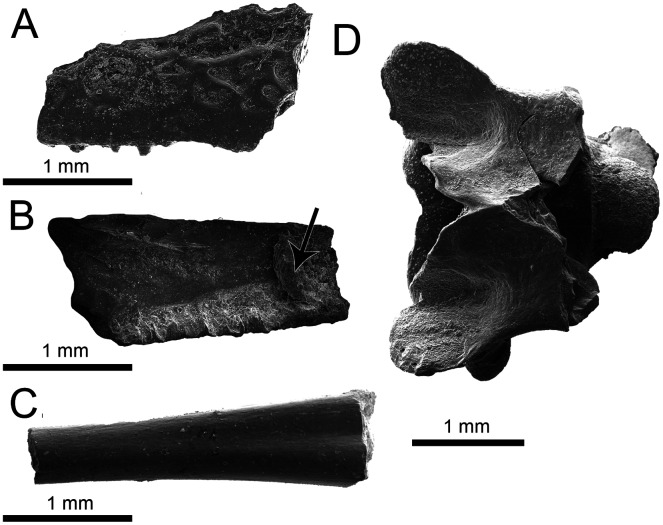
Lissamphibia and Squamata elements. (A–C) Isolated elements of Anura, indet. (A–B) UA-2016-13-145, partial left maxilla in labial (A) and lingual (B) arrow indicates possible pterygoid process. (C) Fused tibia-fibula (UA-2016-13-155). (D) UA-2016-13-295, Squamata indet., vertebra in dorsal view. Scale bars = one mm.

**Description and comments.** UA-2016-13-145 (Figs. 4A, 4B), a partial left maxilla, bears ornamentation in the form of round and irregularly-shaped pits on the dorsolateral surface, becoming smooth inferiorly, toward the alveolar margin. Lingually, alveoli and bits of teeth representing eight or nine tooth positions are present, decreasing in height toward the posterior margin of the element. Anteriorly, a prominence on the lingual face of the maxilla may represent the base of the pterygoid process. A number of fragmentary postcranial elements of frogs are present in the collection, including the characteristic fused epipodial bones. Of these, UA-2016-13-155 (Fig. 4C), a partial tibia/fibula, is representative of Anura.

SQUAMATA Oppel, 1811

Family, genus, and species indet.

**Referred material**. UA-2016-13-295, vertebra missing posterior part of neural arch.

**Description and comment**. This procoelous vertebra bears a round posterior condyle that is constricted at its contact with the centrum (Fig. 4D). The specimen possesses posterior zygapophyses that are broad and angled, and a neural arch apparently with a deep anterior notch that extends to the base of the neural spine. The neural spine and anterior zygapophyses are missing. There is no evidence of zygosphenes and the synapophysis is tall.

SCINCOMORPHA Camp, 1923

SCINCOIDEA Gray, 1825.

**Taxonomic note.** Gray (1825, p. 201) spelled his then-new family “Sincidae,” consistent with his spellings of “*Sincus*” (Daudin, 1802) and *S*. “*sincus*” (Linnaeus, 1758), despite the fact that those authors used the familiar spelling *Scincus*, to which Stark (1828) and subsequent authorities reverted without comment.

PARAMACELLODIDAE Estes, 1983

**Taxonomic note.** Richter (1994) identified tooth crown characteristics for *Paramacellodus* and Paramacellodidae, and this has been followed by Milner & Evans (1998), Evans & Chure (1999), and Evans & Searle (2002). Our concept of the family follows Evans & Chure (1999).

*Sciroseps pawhuskai* gen. et sp. nov.

**Holotype.** UA-2016-13-294, partial left mandible.

**Hypodigm.** The holotype only.

**Type locality and horizon.** The holotype was collected from the Briar site, ca. 18 km NNW Nashville, Howard County, Arkansas (34.09°N, 93.88°W); upper Holly Creek Formation (Lower Cretaceous: Albian).

**Etymology.** The genus name is Latinized from the Greek Skiros (gypsum) + Sepos (lizard or snake, and a commonly used root for lizard names), “gypsum lizard,” an allusion to provenance of the holotype, a gypsum mine. The species is named for Pawhuska (ca. 1763–1809), a chief of the Osage people native to the region, who reportedly got his name (“white hair”) from a powdered wig he acquired during St. Clair’s Defeat (1791).

**Figure 5:**
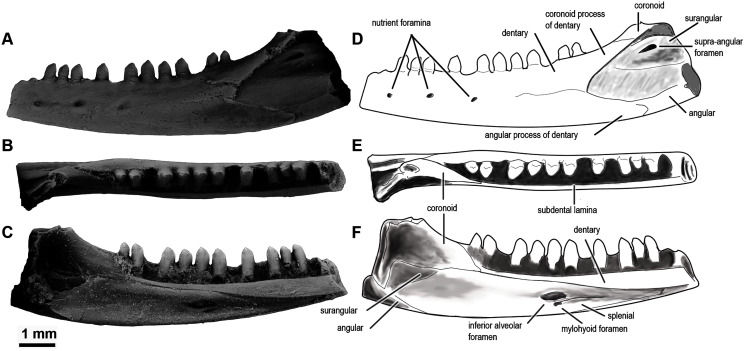
*Sciroseps pawhuskai*, gen et sp. nov. (Scincomorpha: Paramacellodidae). UA-2016-13-294, holotype, left mandible in lingual (A), dorsal (B), and buccal (C) views. (D–F) line drawing of the holotype, highlighting specific skull elements in lingual (D), dorsal (E), and buccal (F) views.

**Diagnosis.** Scincoid broadly similar to Jurassic–Cretaceous lizards of “paramacellodid-cordylid grade” (*sensu* Nydam, 2013a) in tooth morphology and most closely comparable to *Pseudosaurillus* Hoffstetter, 1967 and *Paramacellodus* (*e.g*., *P*. *oweni* Hoffstetter, 1967). Differs from the classic Late Jurassic–Early Cretaceous European paramacellodids *Paramacellodus (P. oweni* Hoffstetter, 1967; holotype NHMUK R. 8131–8132, *Becklesius (B. hoffstetteri* Seiffert, 1975; holotype FUB Gui. A. 56), and *Pseudosaurillus* (*P. becklesi* Hoffstetter, 1967; holotype NHMUK R. 8095) in having a more gracile dentary, less robust and dorsoventrally shorter anterior portion of the subdental lamina, and tooth crowns lacking well defined lingual striae (Fig. 5). *Sciroseps* differs further from *Paramacellodus* and *Pseudosaurillus* in having a more convex dentary dorsoventrally, whereas *Paramacellodus* and *Pseudosaurillus* have nearly straight dentaries. It differs further from *Paramacellodus* in having a more gracile dentition, tooth attachments tending to subpleurodont rather than pleurodont, tooth crowns more spatulate, crista mesialis and crista distalis of posteriormost teeth paraequivalent in length, pars furcata wider, and a smaller crista intercuspidalis. *Sciroseps* differs from *Pseudosaurillus* in dentary having a more acutely pointed apex of the surangular notch, angular clasping of the surangular process of the dentary, coronoid process less exposed laterally between coronoid and surangular, tooth crowns more triangular, tooth crowns extending further beyond lateral parapet of dentary, and presence of well-defined inferior alveolar foramen in splenial. It further differs from *Becklesius* in tooth shafts being more robust and tooth crowns more widely spaced. Among the North American lizards of paramacellodid-cordylid grade, it differs from *Atokasaurus* (*A*. *metarsiodon* Nydam & Cifelli, 2002; holotype OMNH 60535) in having taller, more acutely pointed tooth crowns. *Sciroseps pawhuskai* differs from *Paramacellodus keebleri* Nydam & Cifelli, 2002; OMNH 60576, in lacking a distinct cusp at the angulis mesialis. It differs from *Dakotasaurus* (*D. gillettorum* Nydam, 2013b; holotype MNA V9110) in shorter posterior teeth in dentary, less triangular and more widely spaced tooth crowns. *Sciroseps* differs from *Webbsaurus* (*W. lofgreni* Nydam, 2013b; holotype MNA V10031) in having a convex dentary and more gracile teeth with pointed tooth crowns. Based on these noted distinctions, and the standard practice of naming paleontological material based on phenotypic characteristics (Estes, 1975; Evans & Chure, 1999; Nydam, 2000, 2013b, 2013a; Nydam & Cifelli, 2002; Augé, 2003; DeMar & Breithaupt, 2006; Smith, 2006; Apesteguía, Gómez & Rougier, 2014; Caldwell et al., 2015; Albino, Carrillo-Briceño & Neenan, 2016; Chiarenza & Cau, 2016; Whiteside & Duffin, 2017; Hsiou et al., 2019), the material warrants a new genus: *Sciroseps* and species: *pawhuskai*.

**Description and comments**. The holotype, UA-2016-13-294 (Fig. 5), is a partial left mandible (length = 8.4 mm) with 10 teeth on the dentary and six additional tooth positions preserved. The dentary is missing the symphysial portion. The superior and posterior portions of the coronoid are broken away and the mandible posterior to the coronoid process, including the articular and much of the surangular and angular, is missing (Fig. 5). These imperfections notwithstanding, the specimen is one of the most complete mandibles of a non-polyglyphanodontid lizard from the Cretaceous of North America and is, to our knowledge, the most complete lizard jaw from the Trinity Group.

Salient observations on the dentition are given in the diagnosis and need not be repeated, with the exception that basal replacement/resorption pits are present at the bases of most of the preserved teeth. Here we add only the comment that the teeth are readily distinguished from those of known lizards from both the Cloverly Formation and Trinity Group, with one exception: they closely resemble the single complete tooth preserved in OMNH 60,849, a dentary fragment described by Nydam & Cifelli (2002), fig. 8D under the heading cf. “Paramacellodidae.”

The dentary is comparable in depth to other known paramacellodids and is slightly bowed (convex) ventrally (Figs. 5A, 5C, 5D, 5F). Lingually, the dentary bears a short but conspicuous subdental lamina with a superior margin that forms the medial border of a well-defined subdental gutter (Fig. 5C, 5F). There are three nutrient (mental) foramina preserved on the anterior half of the convex lateral surface (Fig. 5A). The coronoid and angular processes of the dentary are separated by a deep, pointed surangular notch that extends anteriorly to the level of the posteriormost tooth position. The angular process extends posteriorly to the level of the coronoid dorsal process (Figs. 5A, 5D). The coronoid process of the dentary (as exposed on the lateral surface) is nearly equivalent to, but slightly shorter than, the surangular process of the dentary and overlaps the lateral surface of the anterior (dentary) process of the coronoid. The coronoid is broken, but still preserved is the anterior (dentary) process that has a squamous articulation with the medial surface of the coronoid process of the dentary and terminates anteriorly inferior to the posteriormost tooth position as a wedge between the dentary and splenial. There is a well-defined—albeit partially preserved—medially directed crest of the preserved portion of the posteromedial (splenial) process of the coronoid that overlaps a small medial exposure of the surangular and would have formed a prominent anterior boundary of the adductor fossa. The dorsal (coronoid) process is missing the apex and the posterior (surangular) process of the coronoid is not preserved (Figs. 5B, 5E). Lingually, the splenial closes the Meckelian fossa for nearly the entire length of the dentary, but terminates several tooth positions (count indeterminate, but likely five to seven) short of the symphysis. Posteriorly the splenial terminates just anterior to the level of the medial process of the coronoid. A large anterior inferior alveolar foramen is completely enclosed within the splenial and is directly superior to a small, single anterior mylohyoid foramen. The anterior portions of the surangular and angular are preserved in articulation with the dentary. On the external surface of the specimen the surangular fills all but the inferior most aspect of the surangular notch, which extends anteriorly to the level of the posteriormost preserved tooth. The internal extent of the surangular is obscured by the overlapping dentary, coronoid, and splenial. A prominent anterior supra-angular foramen is present superiorly on the lateral surface of the surangular, inferior to the coronoid process of the coronoid. The angular weakly clasps the surangular process of the dentary.

Morphologically, *Sciroseps pawhuskai* can be characterized as a scincoid of paramacellodid-cordylid grade, which made their North American appearance in the Late Jurassic and persisted through to the late Campanian in what is now the Western Interior (Nydam, 2013a, 2013b). Given that paramacellodid-cordylid grade lizards are dominant elements of known squamate assemblages from the Aptian–Albian Cloverly Formation and Trinity Group (Nydam & Cifelli, 2002), the presence of *Sciroseps* in the Holly Creek Formation is unsurprising, although it is a welcome addition to knowledge.

TESTUDINATA Klein, 1760

Family, genus, and species indet.

**Taxonomic note**. We follow most contemporary authorities in attributing Testudinata to Klein (1760); see discussion in (Joyce, Parham & Gauthier, 2004). The name was proposed in his earlier work (Klein, 1751), and is cited as such in the influential treatment by Oppel (1811). However, Klein’s original (1751) work was published before Linnaeus’ (1758) classification. See also extended discussion by Dubois & Bour (2010).

SOLEMYDIDAE De Lapparent De Broin & Murelaga, 1996

*Naomichelys speciosa* Hay, 1908

**Referred material:** UA-2016-13-001 to UA-2016-13-13; UA-2016-13-259; UA-2016-13-325.

**Figure 6:**
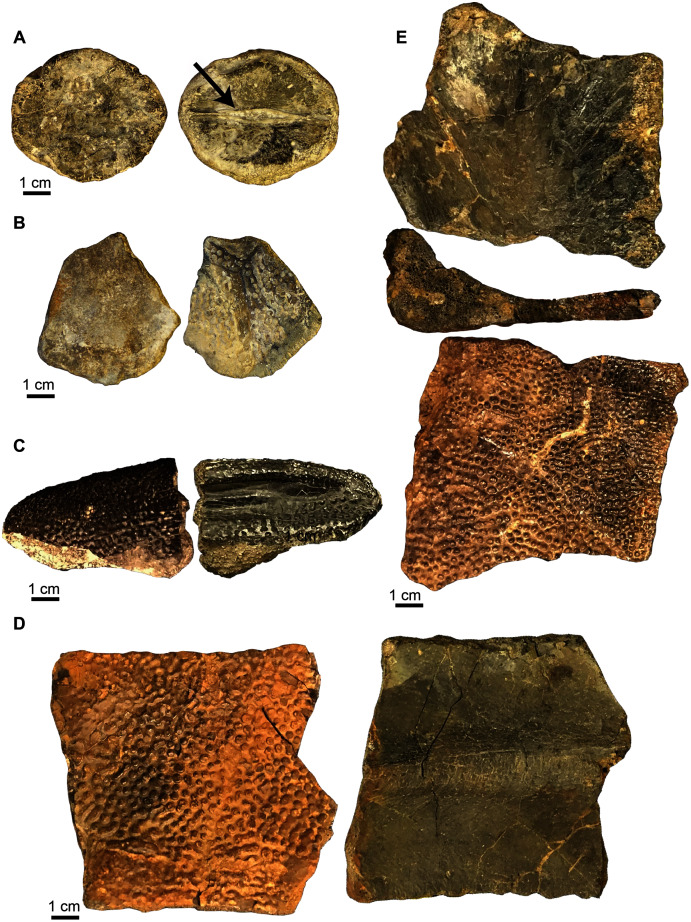
*Naomichelys speciosa* carapace elements. (A) First neural bone (UA-2016-13-9) in dorsal (left) and ventral views; note bifurcating ridge down the center of the bone (arrow) in ventral view (right). (B) Nuchal fragment (UA-2016-13-12) in dorsal (left) and ventral (right) views. (C) Unusually shaped marginal fragment (UA-2016-13-7). (D) Thick costal fragment (UA-2016-13-4). (E) Unusual costal or marginal fragment (UA-2016-12-1) in ventral (top), transverse (middle; note L-shaped cross section) and dorsal (bottom) views. Scale bars = one cm.

**Figure 7:**
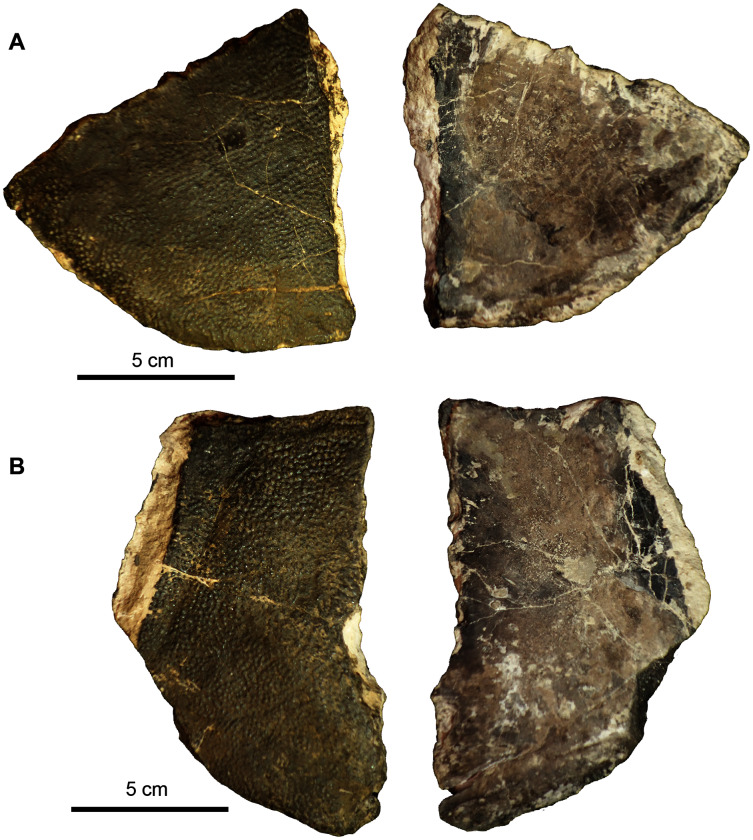
*Naomichelys speciosa* plastron fragments. (A) Right xiphiplastron (UA-2016-13-3) in ventral (left) and dorsal (right) views. (B) An unknown fragment (UA-2016-13-2) in ventral (left) and dorsal (right) views. Scale bars = five cm.

**Description and comments**. Several large carapace and plastron fragments as well as smaller carapace fragments from screen-washing were recovered from the drainage ditch at the Briar Site (Figs. 6, 7). This material is readily identified by the classic pustulate ornamentation characteristic of *Naomichelys speciosa*. Given the geographic proximity of a large and complete specimen from the coeval Antlers Formation of Texas (Joyce, Sterli & Chapman, 2014), we attribute the Holly Creek material to *Naomichelys speciosa* with little doubt.

***Carapace fragments*.** The carapace is represented by several fragments and six identifiable elements. These include the first neural bone (Fig. 6A), a nuchal fragment (Fig. 6B), an unusually shaped marginal fragment (Fig. 6C), one thick costal fragment (Fig. 6D), and an L-shaped fragment in cross-section (Fig. 6E) that we tentatively identify as a marginal scute. The carapace bones are very thick, ~1.5 to 2 cm in thickness. The first neural is hexagonal in shape and bears a proximo-distal ridge that bifurcates in the center of the ventral side, where it forms a long groove (see arrow in Fig. 6A). The nuchal (Fig. 6B) is trapezoidal in shape and is broken in half with half missing.

***Plastron fragments*.** Two large plastron fragments were recovered. This includes a right xiphiplastron (Fig. 7A) and an unknown fragment, possibly a hypoplastron, mesoplastron, or epiplastron (Fig. 7B).

An additional small piece of shell (not figured) found within the microvertebrate assemblage, approximately two mm tall is here identified as belonging to the Cretaceous genus *Naomichelys*. This oblong piece of shell contains five of the characteristic pustules, diagnostic of the clade Solemydidae (De Lapparent De Broin & Murelaga, 1996; Joyce et al., 2011; Joyce, Sterli & Chapman, 2014). These pustules are relatively widely-spaced and are approximately 0.4 mm in diameter, smaller than those described from specimens from the Trinity Group (0.75–2 mm; Joyce, Sterli & Chapman, 2014) and Cloverly Formation (0.5–1.2 mm; Ostrom, 1970). Based on their size and spacing, it is possible that this piece comes from the periphery of a young individual.

CRYPTODIRA Cope, 1868

TRIONYCHOIDEA Fitzinger, 1826

Genus and species indet.

**Referred Material.** UA-2016-13-080.

**Figure 8:**
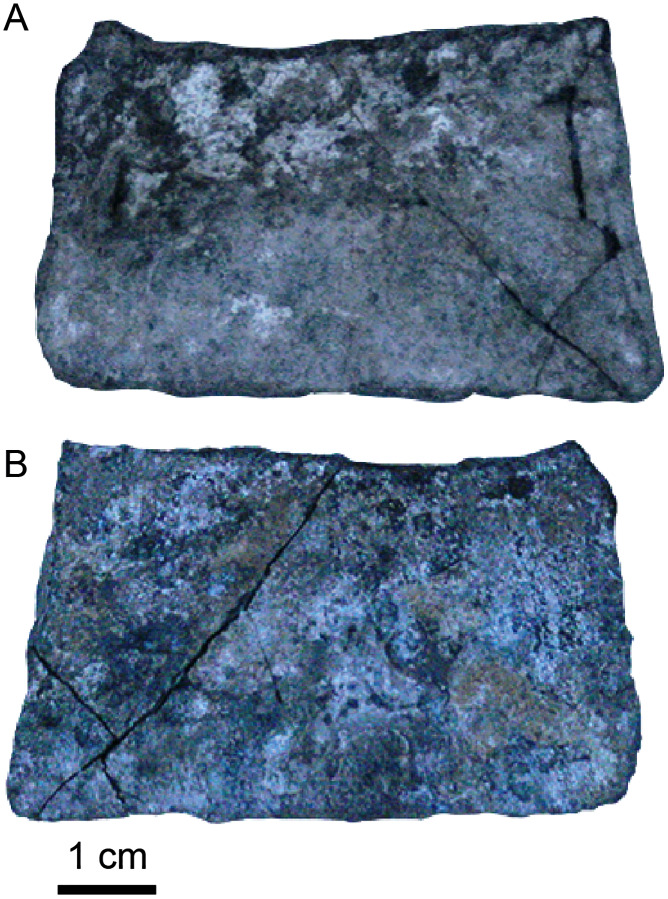
Trionychidae costal fragment. UA-2016-13-80 in (A) ventral and (B) dorsal views. Scale bar = one cm.

**Description and comments**. One costal carapace fragment was recovered from the site. It is poorly preserved, and identification of the exact costal bone is not possible (Fig. 8). The bone is 6.53 cm long but is broken at the distal end and 3.86 cm wide. There is a protrusion of the proximal end of the rib (left) that is 0.6 cm wide. The surface is smooth with no ornamentation. Identification beyond a trionychoid turtle is not possible given the quality of the material.

ARCHOSAURIA Cope, 1870

CROCODYLIFORMES Hay, 1930

Family, genus, and species indet.

**Referred material.** UA-2016-13-16 to UA-2016-13-24; UA-2016-13-29 to UA-2016-13-37; UA-2016-13-41; UA-2016-322 to UA-2016-324.

**Description and comments.** A significant amount of material recovered from the Briar Site belongs to crocodylomorphs. This includes cranial elements, vertebrae, and osteoderms.

**Figure 9:**
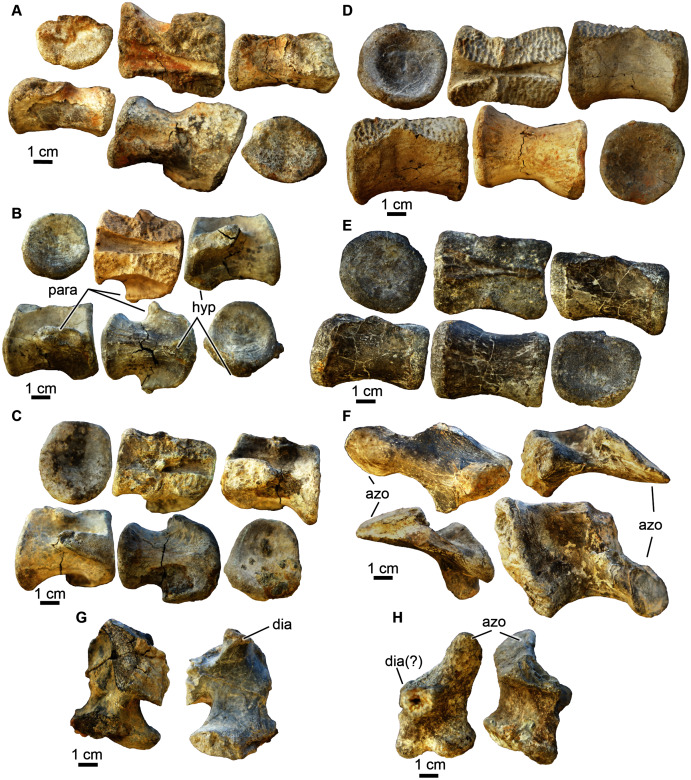
Crocodyliformes vertebrae. (A) A possible sacral vertebra (UA-2016-13-29) in (top row, left to right) anterior, dorsal, right lateral views; and (second row, left to right) in left lateral, dorsal, and posterior views. (B) Cervical vertebra (UA-2016-13-41) in (top row, left to right) posterior, ventral, and left lateral view; and (second row, left to right) right lateral, ventral, and anterior views. (C) Cervical vertebra (UA-2016-13-31) in (top row, left to right) posterior, dorsal, and left lateral views; and (second row, left to right) in right lateral, ventral, and posterior views. (D) Dorsal vertebra (UA-2016-13-32) in (top row, left to right) anterior, dorsal, and right lateral views; and (second row, left to right) left lateral, ventral, and posterior views. (E) Dorsal vertebra (UA-2016-13-30) in (first row, left to right) posterior, ventral, and right lateral views; and (second row, left to right) left lateral, dorsal, and anterior views. (F) Ascending process of a neural arch preserving a left prezygapophysis (UA-2016-13-35) in (top row, left to right) left lateral and anterior views; and in (bottom row, left to right) posterior and medial views. (G) Ascending process of a neural arch preserving a portion of the diapophysis (UA-2016-13-36) in medial (left) and lateral (right) views. The pre-and post zygapophyses are not preserved. (H) Ascending process of a neural arch preserving the right prezygapophysis (UA-2016-13-37) in (left to right) right lateral and posterior views. Azo, prezygapophysis; dia, diapophysis; hyp, hypapophysis; para, parapophysis. Scale bars = one cm.

***Vertebral elements***. One possible sacral vertebra centrum, two cervical vertebral centra and four dorsal vertebral centra are preserved within the assemblage. All are amphicoelous (Fig. 9). None have fused neural processes and the neural processes were clearly un-sutured to the centra. The possible sacral centrum is wider posteriorly than anteriorly and is dorsoventrally compressed (Fig. 9A). There is a distinct groove down the center of the vertebra when viewed ventrally, making the distal margins of the centrum look double-lobed. The parapophyses occur distally on the centrum (Fig. 9A). The two cervical vertebrae have distinct parapophyses and a blunted hypapophysis that together form a triangular shape when seen in ventral view (Figs. 9B, 9C). There is a distinct medial carina that extends from the hypapophysis through the midline of the centrum in ventral view and terminates in front of the posterior margin of the vertebra (Fig. 9C). The two are relatively stout and are almost as wide as they are long. The four dorsal vertebrae are hourglass-shaped when viewed dorsoventrally and are longer than they are wide (Figs. 9D, 9E). Neural processes were also preserved (Figs. 9F–9H) as separate fragmentary elements, and one vertebra has its associated neural processes (See videos of this more complete vertebra in Supplemental Files: Video S1 (https://doi.org/10.17602/M2/M369562) and Video S2 (https://doi.org/10.17602/M2/M369565)). Two fragmentary elements preserve the ascending process of the neural arch, neural arch suture, and a prezygapophysis (Figs. 9F, 9H). UA 2016-13-036 (Fig. 9G) preserves an ascending arch of the neural arch and a diapophysis but no distinct pre-or postzygapophyses are preserved. The vertebra shown in the supplemental videos is incomplete, but has the pre-and postzygapophyses, and a transverse process on one side. Video S1 shows this vertebra as scanned from the original specimen, and Video S2 shows a reconstruction with the postzygapophyses and neural process in life position. There is clear distortion of the neural arches suggesting burial alteration.

**Figure 10:**
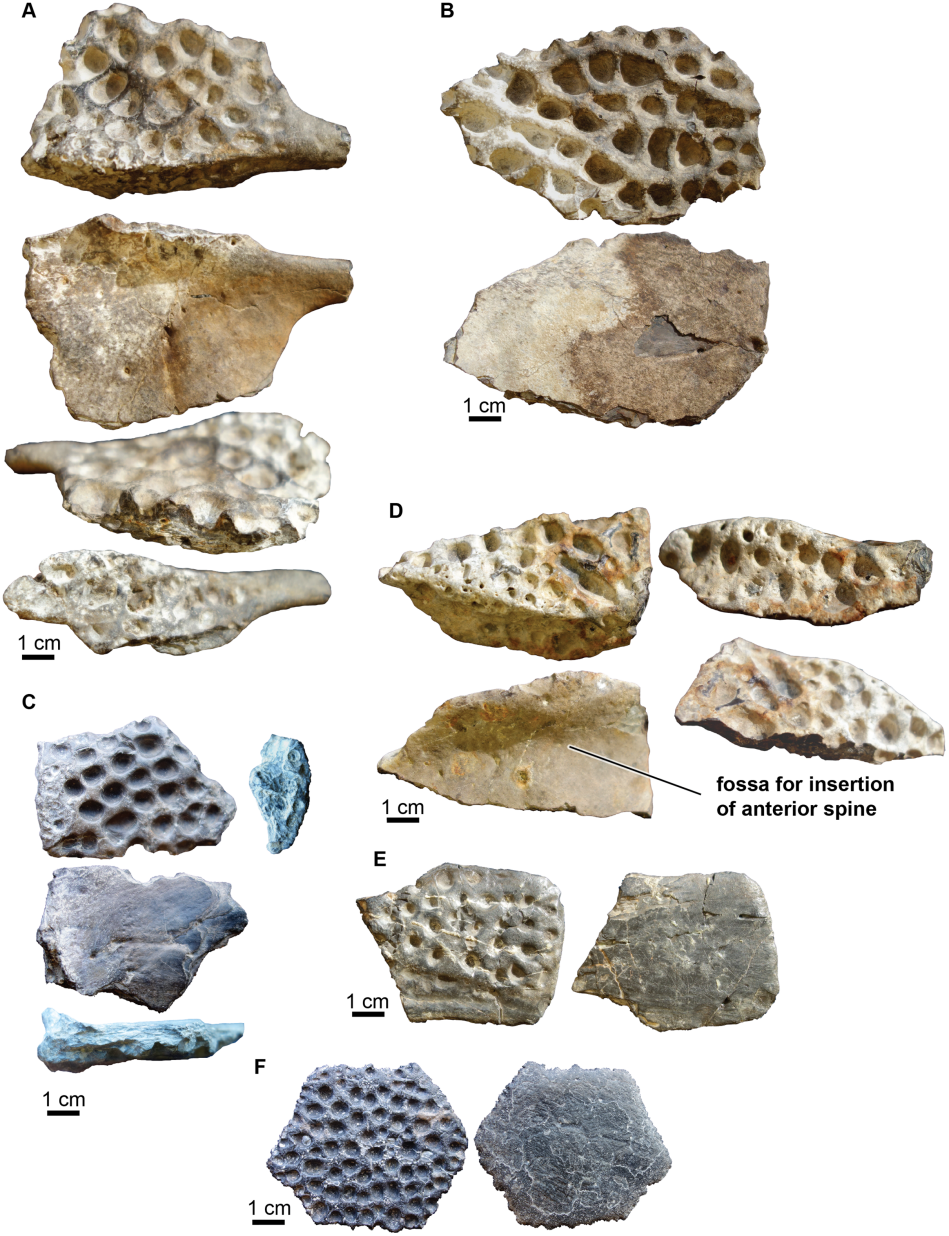
Crocodyliformes osteoderms. (A–D) Dorsal osteoderms and (E–F) ventral osteoderm. (A) Dorsal osteoderm (UA-2016-19) in (top to bottom) dorsal, ventral, medial, and lateral views. (B) Fragment of a dorsal osteoderm (UA-2016-13-18) in (top to bottom) dorsal and ventral views. (C) Fragment of a dorsal osteoderm (UA-2016-13-27) in (top to bottom) dorsal, ventral, transverse, and (second column) medial views. (D) Dorsal osteoderm (UA-2016-13-24) in (top row, left to right) dorsal, lateral(bottom row, left to right) ventral, and medial views. A distinct groove or fossa is evident for insertion of the anterior spine (*e.g.*, observed in A). (E) A ventral osteoderm (UA-2016-13-22) in ventral and dorsal views. (F) A central ventral osteoderm (UA-2016-13-23) in ventral and dorsal views. Scale bars = one cm.

***Osteoderms***. Four partial dorsal osteoderms were preserved. All are ornamented with round pits on the dorsal side and are smooth on their ventral side (Figs. 10A–10D). One osteoderm (Fig. 10A) preserves a portion of the forward projecting spine that originates on the anterolateral corner (Martin, Delfino & Smith, 2016). It is smooth with no ornamentation. Martin, Delfino & Smith (2016) describe these processes as inserting into a distinct groove on the posterolateral corner of the anteriorly adjacent osteoderm. This insertion is a smooth, concave surface or fossa when seen in ventral view and can be seen on one of the osteoderms (Fig. 10D). On the dorsal side of one of the osteoderms there is an anteroposterior carina that runs lateral to the midline of the osteoderm (dorsal surface of Figs. 10A, 10D).

Two ventral osteoderms are preserved (Figs. 10E, 10F). One is hexagonal in shape (Fig. 10F) while the other is fragmentary and is more rectangular in outline (Fig. 10E). All ventral osteoderms are smooth on their dorsal surfaces and ornamented with large circular pits on their ventral side. Martin, Delfino & Smith (2016) describe similar hexagonal-shaped ventral osteoderms as the most central osteoderm on the ventral shield of the coelognathosuchian *Anteophthalmosuchus*. Marginal ventral osteoderms are not hexagonal but four-to five-sided (Martin, Delfino & Smith, 2016), suggesting that osteoderm UA-2016-13-22 (Fig. 10E) is from the margin of the ventral shield and UA-2016-13-22 (Fig. 10F) is a central ventral osteoderm. The hexagonal osteoderm from the Arkansas material is un-sutured, suggesting this individual was a sub-adult.

Unfortunately, the described crocodyliform postcranial material was not found associated with identifiable cranial material (see below). The proximity of the Holly Creek Formation to the Twin Mountains Formation suggests the material may be attributed to the cranial elements of *Paluxysuchus newmani* Adams, 2013. However, since material was not found associated with the cranial elements (see below), we conservatively refer to the osteoderms and vertebrae to Crocodyliformes.

NEOSUCHIA Benton & Clark, 1988

COELOGNATHOSUCHIA Martin et al., 2014

*Paluxysuchus newmani* Adams, 2013

**Referred material.**; UA-2016-13-25; UA-2016-13-27; UA-2016-13-165, UA-2016-13-169, 37 partial to complete teeth; UA-2016-13-172; TMM 41881.

**Description and comments.** Two skull elements, teeth, and one skull have been preserved in the site. Cranial material can be directly compared to cranial material of *Paluxysuchus newmani*, a crocodyliform described from the correlative and geographically close Twin Mountain Formation.

***Cranial Elements*.**

**Figure 11:**
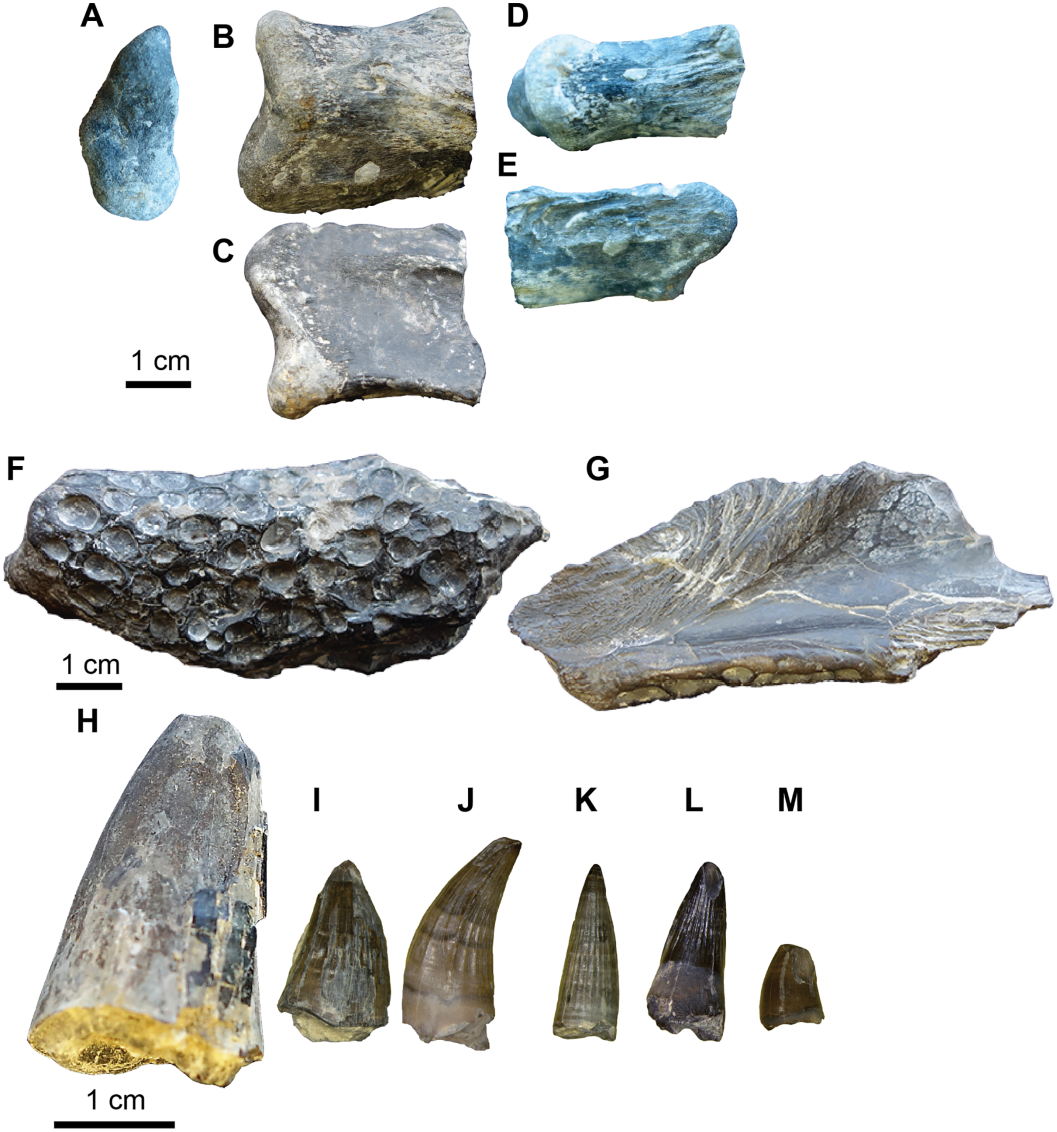
*Paluxysuchus* (Crocodylia: Coelognathosuchia), cranial elements. (A–E) Right quadrate (UA-2016-13-27) in posterior (A), dorsal (B), ventral (C), medial (D), and lateral (E) views. (F–G) Posterior lobe of the left quadratojugal (UA-2016-13-25) in dorsal (F), and ventral (G) views. (H–M), teeth (UA-2016-13-63). Scale bars = one cm.

**Figure 12:**
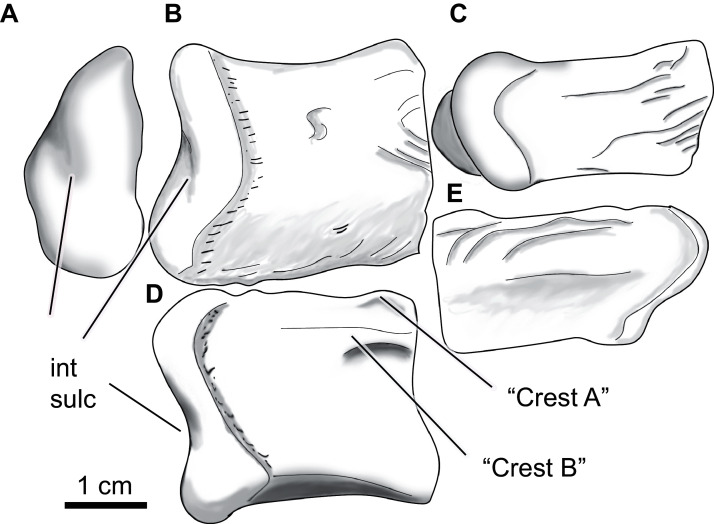
*Paluxysuchus* (Crocodylia: Coelognathosuchia), right quadrate. UA-2016-13-27 in posterior (A), dorsal (B), ventral (C), medial (D), and lateral (E) views. “Crest A” and “B” as described by Adams (2013) are observed, with “Crest B” being the most defined crest. int sulc = intercondylar sulcus. Scale bar = one cm.

The posterior end (mandibular condyle) of a right quadrate (Figs. 11A–11E) is preserved, as is a left quadratojugal. The lobe-shaped posterior region of the quadratojugal is preserved and only part of the plate-shaped anterior region is present (Figs. 11F–11G). The posterior region is highly sculpted on the dorsal side, with rounded pits that are ~0.5 cm in diameter and up to ~0.3 cm deep (Fig. 12A). The ventral side of the posterior quadratojugal is concave with a rugose medial margin and deep groove for contact with the jugal (Fig. 12B).

**Figure 13:**
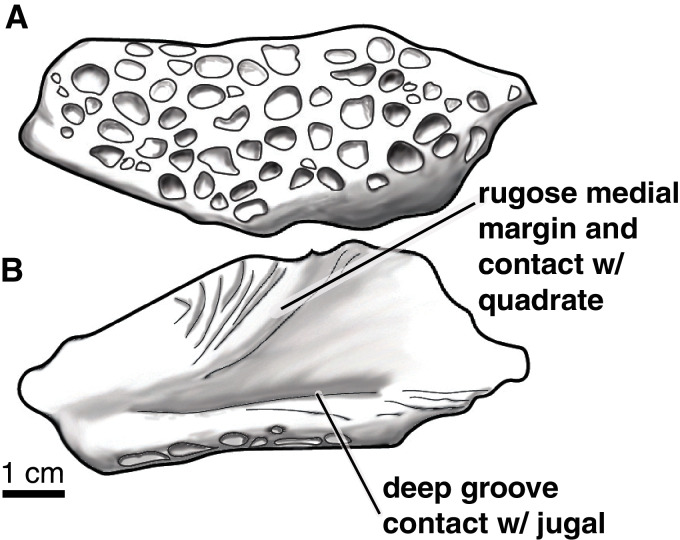
*Paluxysuchus* (Crocodylia: Coelognathosuchia), posterior lobe of the left quadratojugal. UA-2016-13-25 in (A) dorsal, and (B) ventral views. There is a distinct rugose medial margin for contact with the quadrate and a deep groove for contact with the jugal. The dorsal surface is ornamented with deep pits. Scale bar = one cm.

The quadrate has a rugose lateral margin for articulation with the quadratojugal. Only the posterior side of the quadrate is preserved and therefore only the most posterior end of crests “A” and “B” (*sensu* Iordansky, 1973) described by Adams (2013) are observed (Fig. 13D). There is a deep fossa medial to the crest (like that described by Adams, 2013). The lateral side of the quadrate is strongly rugose; this texture is composed of several ridges and fossae that wrap around to the dorsal side of the quadrate (Figs. 13B, 13E). The mandibular condyle is composed of medial and lateral hemicondyles, with the lateral condyle being broad at the lateral margin and shallowing medially. The medial condyle is longer and tapers medially. The two are separated by a deep intercondylar sulcus (Figs. 13A, 13B, and 13D).

**Figure 14:**
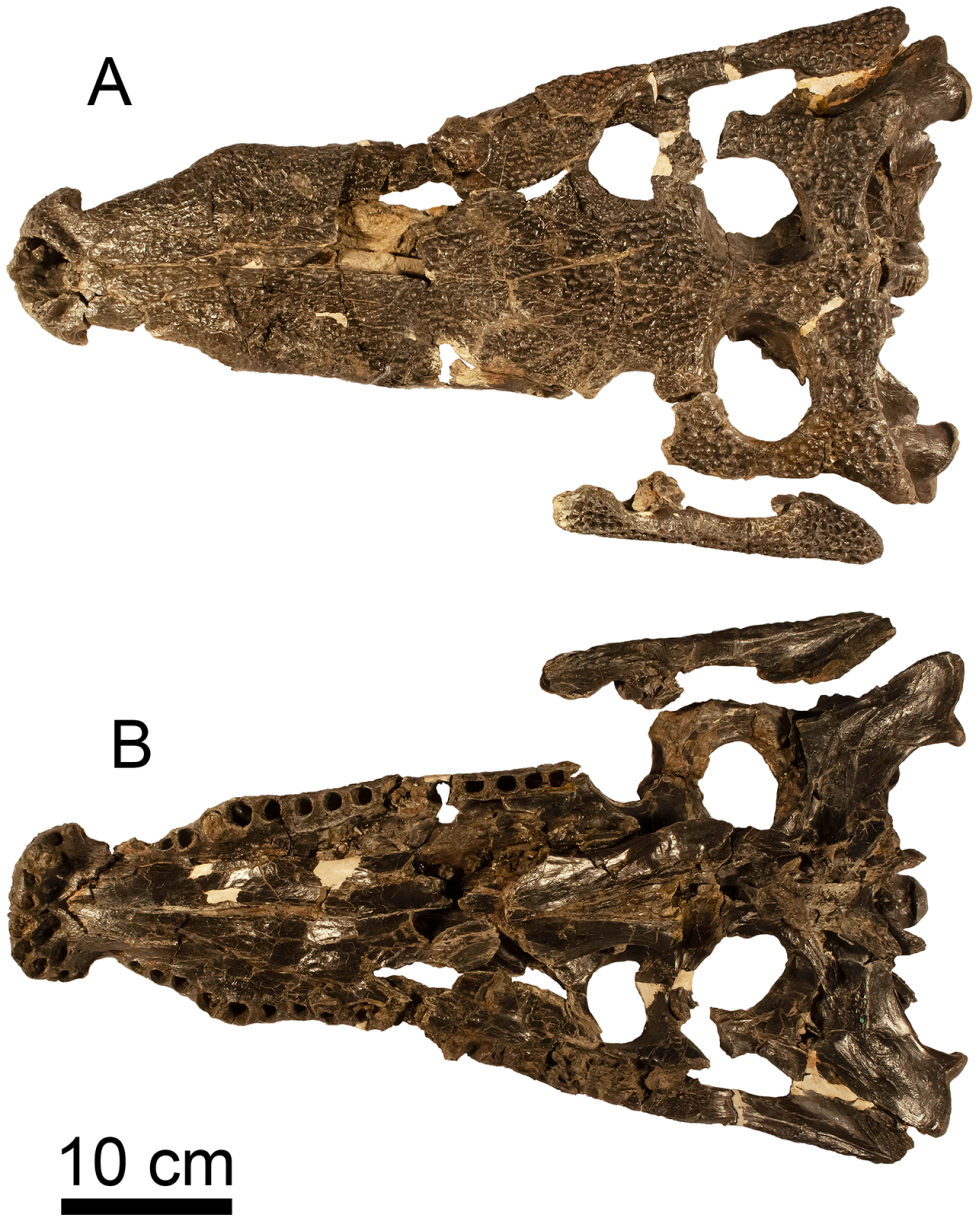
cf. *Paluxysuchus* (Crocodylia: Coelognathosuchia) skull. (A) Dorsal and (B) ventral view of a coelognathosuchian skull (TMM 41,881) from the Briar Site, housed at TMM.

A large skull of a coelognathosuchian was discovered at the site in the early 1970s and provided to Dr. N. F. Williams of the Arkansas Geological Commission (now the Arkansas Geological Survey). This skull was briefly described by Dr. James H. Quinn, former paleontologist at the University of Arkansas along with an ornithomimid foot (Quinn, 1973) that was later named *Arkansaurus fridayi* (Hunt & Quinn, 2018). In 1975, the skull was shared with Dr. Wann Langston of the Texas Memorial Museum at the University of Texas and until recently has gone unstudied. This skull, TMM 41,881, is 53 cm long (measured from the occipital condyle) by 30 cm wide at the base of the skull. The dorsal surface is highly ornamented with rounded pits (Fig. 14). No teeth are preserved within the tooth sockets of the skull. The quadrate and quadratojugal of this specimen are larger than the isolated elements described above. Additional description of this material will be required for adequate taxonomic placement and will be the subject of future study. As such we figure this specimen for sake of completion, but do not go into further detail.

Straight to recurved teeth have large carinae with fine striations radiating apically along the crown. The largest individual of the microvertebrate screen-washed material is represented by a tooth that is 10.1 mm long, though most fall within a range of 1–2 mm in crown height. Similar teeth have long been known from the Cloverly Formation (Ostrom, 1970), Trinity Group (Langston, 1974; Cifelli et al., 1997a), Cedar Mountain Formation (Cifelli et al., 1997b, 1999b; Kirkland et al., 1999; Frederickson et al., 2017), and Arundel Clay (Lull, Clark & Berry, 1911, plate 20, Fig. 7; Frederickson, Lipka & Cifelli, 2018). Larger teeth (not found in screen-washed material), also include several conical to slightly recurved teeth bearing large carinae, with fine striations radiating apically along the crown (Figs. 9H–9M). Much of the microvertebrate material is also composed of coelognathosuchian teeth and likely can be ascribed to juveniles of the macrovertebrate coelognathosuchian.

We assign coelognathosuchian cranial material from the Briar Site to *Paluxysuchus newmani* Adams, 2013 based on close similarity in known craniodental anatomy to the holotype, SMU 76602. This includes a quadrate that is unsculpted except for the lateral margin and a ventral surface that is smooth except for the deep fossa between the strongly developed crest B and weak crest A. The horizontally aligned medial and lateral condyles are also similar to those of *Paluxysuchus*. This is very different from *Deltasuchus*, a much younger crocodyliform from the Cenomanian Woodbine Formation of Texas (Adams, Noto & Drumheller, 2017). The quadrate of *Deltasuchus* has a foramen aëreum, is heavily rugose on its dorsal surface and has weakly developed crests A and B. The medial hemicondyle is angled relative to the horizontally aligned lateral hemicondyle. The quadratojugal discovered at the Briar Site is also lobe-shaped in the posterior region and its ventral surface is also concave with a rugose medial margin, similar to the quadratojugal of *Paluxysuchus*. The size disparity between the quadratojugal and the quadrate as well as the presence of the TMM 41881 skull suggests that at least three individuals of *P. newmani* are preserved at the site. Presence of this taxon in the Holly Creek Formation is unsurprising, given its presence in the penecontemporaneous Twin Mountains Formation (Trinity Group) of Texas.

cf. BERNISSARTIIDAE Dollo, 1883

Family, genus, and species indet.

**Taxonomic note.** Spelling of the family-group name is “Bernissartidae” in the original publication (Dollo, 1883, p. 334).

**Referred Material.** UA-2016-13-171; UA-2016-13-173; UA-2016-13-175; UA-2016-13-176; 111 partial to complete teeth.

**Figure 15:**
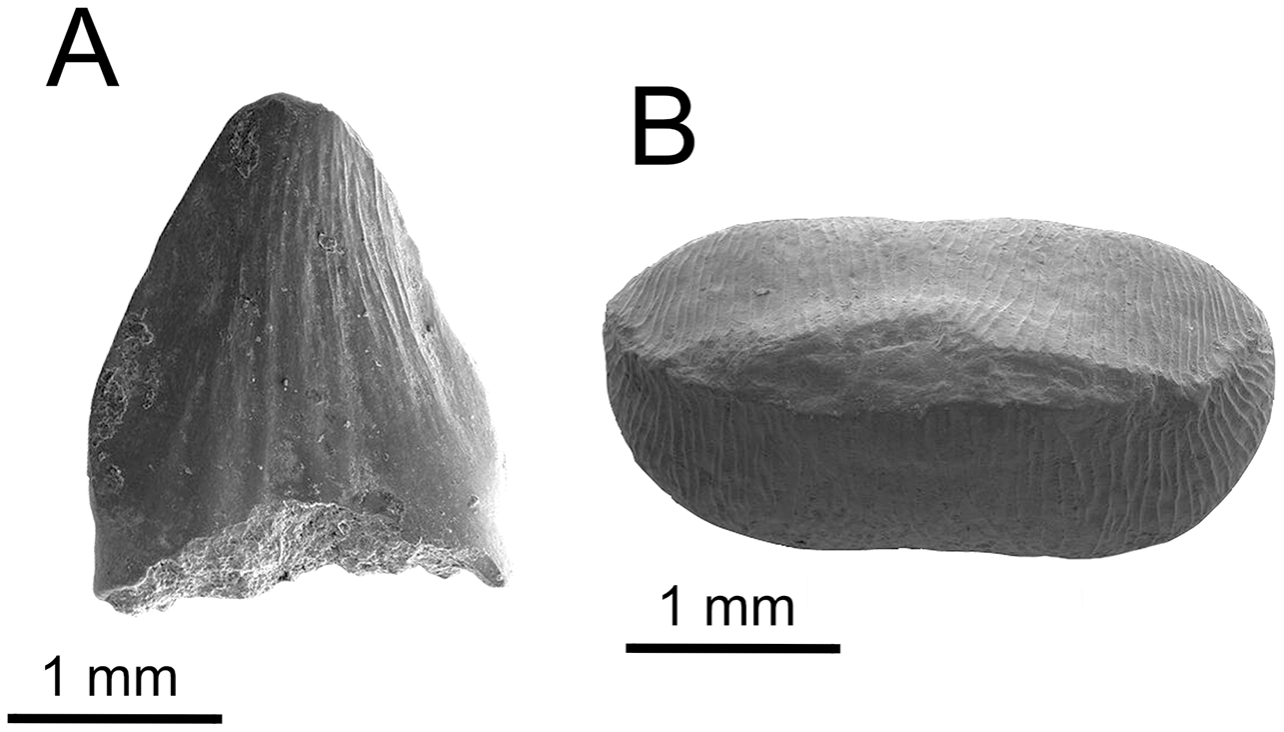
cf. Bernissartiidae tooth. UA-2016-13-175 in (A) lateral and (B) occlusal views. Scale bar = one mm.

**Description and comments.** The largest teeth (up to 7.6 mm in anteroposterior width) are circular or reniform with flattened or rounded occlusal surfaces in labiolingual view, indicating a crushing masticatory function. Smaller teeth are triangular or lunate in labiolingual view; these are likely anterior teeth from the same taxon (or taxa) as the other teeth (Figs. 15A, 15B). As in many other terrestrial microvertebrate assemblages of Albian–Cenomanian age (Garrison et al., 2007), crocodilian teeth are by far the most common tetrapod elements encountered in the sample from the Holly Creek Formation. The most abundant crocodilian teeth come from one or more taxa that, at current resolution, are indistinguishable from those referred to *Bernissartia* (Buffetaut & Ford, 1979, figs. 1–26). Button-shaped bernissartiid teeth have been identified previously from the Trinity Group of Texas (Langston, 1974; Winkler, Murry & Jacobs, 1990), Antlers Formation of Oklahoma (Cifelli et al., 1997a), and Cloverly Formation of Wyoming and Montana (Oreska, Carrano & Dzikiewicz, 2013). These authors further identified tall-crowned triangular teeth as comparing favorably to those of atoposaurids, and similar teeth have been identified from *Wannchampsus kirpachi* Adams, 2014. Tennant, Mannion & Upchurch (2016) recently reviewed the systematics of atoposaurids and concluded that distribution of Atoposauridae is limited to the Middle–Late Jurassic of Europe, making this identification unlikely.

DINOSAURIA Owen, 1842

SAUROPODA Marsh, 1878

MACRONARIA Wilson & Sereno, 1998

TITANOSAURIFORMES Salgado, Coria & Calvo, 1997

BRACHIOSAURIDAE Riggs, 1904

cf. *Sauroposeidon*, sp. indet.

**Reference material.** UA-2016-13-180; UA-2016-13-038; UA-2016-13-076; UA-2016-76a.

**Figure 16:**
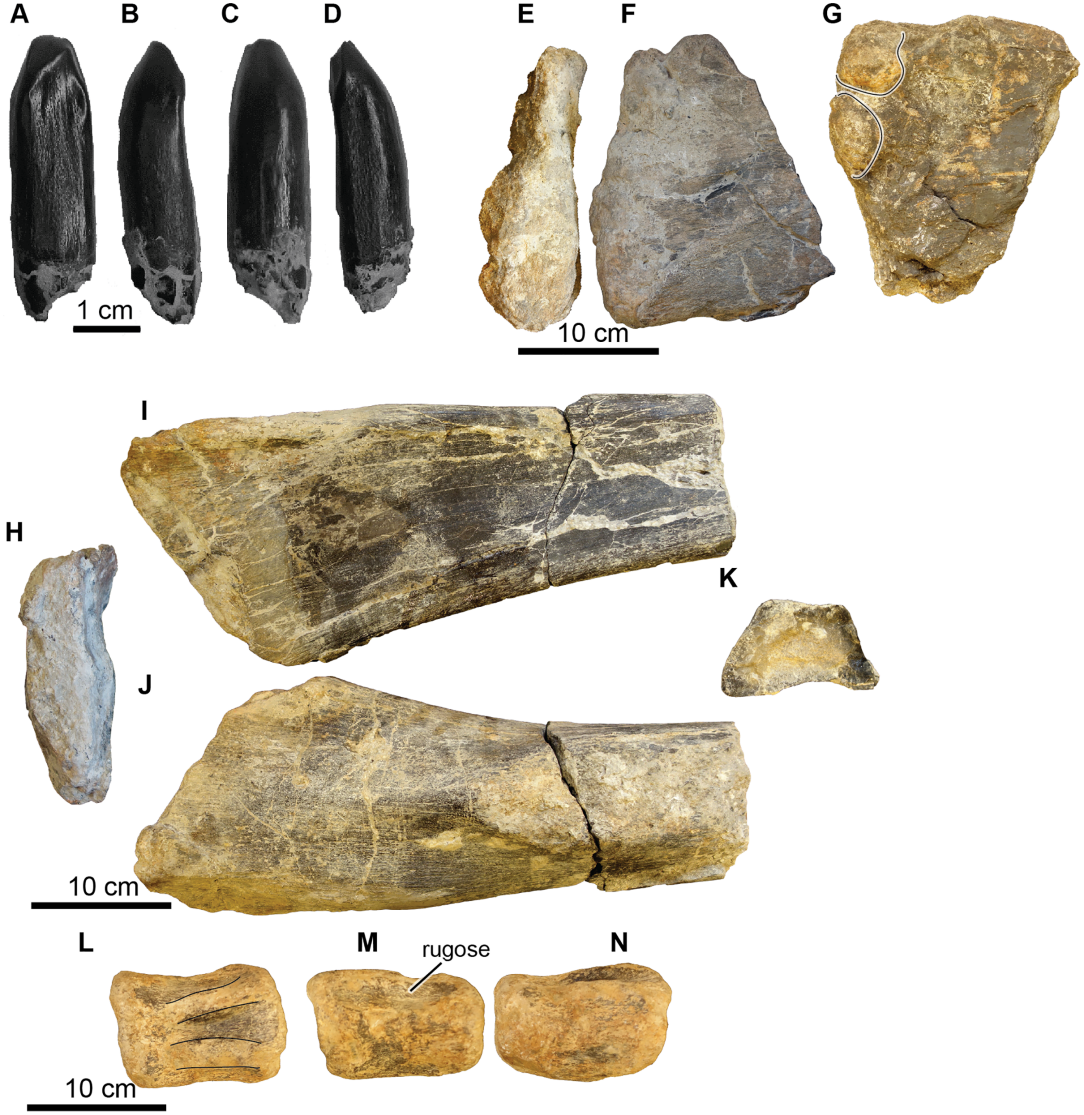
Sauropoda, various elements. (A–D) Brachiosauridae, UA-2016-13-180, tooth in lingual (A), lateral 1 (B), buccal (C), and lateral 2 (D) views. Scale bar = one cm. (E–N), Sauropoda, indet. (E–G) proximal left humerus? (UA-2016-13-76) in distal (E), lateral (F), and medial (G) views. There are two distinct condyles (line-highlighted) in the medial view. Scale bar = 10 cm. (H-K) A right humerus? (UA-2016-13-76a) in proximal (H), medial (I), lateral (J), and distal (K; cross-sectional aspect of broken end) views. Scale bar = 10 cm. (L–N) A middle caudal vertebra (UA-2016-13-38) in dorsal (L; weathered neural processes line-highlighted), right lateral (M) and left lateral (N) views. Scale bar = 10 cm.

**Description and** **comment.** Four elements from a sauropod were recovered from the quarry (Fig. 16). One tooth crown (Figs. 16A–16D) was found. The remaining specimens are parts of postcranial elements and include a probable distal end of a left humerus (Figs. 16E–16G), a possible distal right humerus (Figs. 16H–16K), and a caudal centrum (Figs. 16L–16N).

***Cranial elements*.** The tooth (UA-2016-12-180) from the Holly Creek Formation is parallel-sided (non-spatulate) and approximately 30 mm in crown height, with a slenderness index of 2.7 (crown height/mesiodistal crown width; Upchurch, 1998). Two wear facets are observed on the apical end of the tooth with one larger than the other; both are planar and are angled either mesially or distally. The larger facet is angled ~40° from vertical and the smaller facet is angled ~20° from vertical. The enamel is smooth, and the lingual surface is concave. The tooth crown is similar to that of other titanosauriform sauropods and is similar to those of *Paluxysaurus* Rose, 2007, which is probably a junior subjective synonym of *Sauroposeidon* Wedel, Cifelli & Sanders, 2000 (D’Emic, 2013).

***Postcranial elements*.** A limb fragment, here identified as a distal humerus, is incomplete with both articular ends missing (Figs. 16H–16K). It has a maximum width of 17.9 cm. The cross-section is trapezoidal with a height of 7.3 cm and a width of 12.0 cm. The medial side is slightly concave. The lateral side flattens proximally and widens to the trapezoidal cross-section distally owing to the presence of a distinct rounded ridge that extends the length of the limb fragment. We tentatively identify a large, flattened fragment (Figs. 15E–15G) as a partial proximal end of a left humerus based on a similar cranial view of *Paluxysaurus* (Rose, 2007). Its maximum width at the proximal articular end is 21.0 cm and is 7.5 cm at its thickest point. There are two possible condyles on the proximal-medial surface (Fig. 16G).

The caudal vertebra, which is from the middle of the series, is well-worn (Figs. 16L–16N). The neural groove is present; however, the neural process is worn away and no sutures are preserved. The neural process occupies ~two-thirds of the length of the centrum. The centrum is wider than it is tall, with a maximum width of eight cm and a maximum length of 10.2 cm. Although poorly preserved, the vertebra appears to be amphicoelous. The bone near the lateral side of the neural process is rugose in nature.

Sauropod remains from the Antlers and other named units of the Trinity Group have a complex taxonomic history and the fossils from the Briar Site locality are non-diagnostic, with the exception of the tooth. Teeth and sparse remains from Texas were initially identified to the genera *Astrodon* Johnston, 1859 or *Pleurocoelus* Marsh, 1888 (see Langston, 1974), though both genera are now considered *nomina dubia* (D’Emic, 2013). Currently, four named species are identified from the Aptian/Albian Antlers Formation and Trinity Group: *Cedarosaurus weiskopfae* Tidwell, Carpenter & Brooks, 1999; *Sauroposeidon proteles* Wedel, Cifelli & Sanders, 2000; *Paluxysaurus jonesi* Rose, 2007; and *Astrophocaudia slaughteri* D’Emic, 2013. The tooth from the Holly Creek matches well with the general “*Astrodon”* tooth morphology and is similar to sauropod teeth from the Aptian/Albian of Texas (Rose, 2007; D’Emic, 2013). The tooth from the Holly Creek Formation lacks the longitudinal lingual groove seen in *Astrophocaudia slaughteri* and is fairly similar to those described for *Paluxysaurus jonesi* by Rose (2007, fig 6), as *Camarasaurus*-like but less spatulate. D’Emic (2013) synonymized *P. jonesi* with *Sauroposeidon proteles*, recovering both as basal members of Somphospondyli. By contrast, *Sauroposeidon* was originally referred to Brachiosauridae (Wedel, Cifelli & Sanders, 2000; Naish et al., 2004; Upchurch et al., 2004). The Holly Creek material offers no insights into this debate; however, given the morphology of the teeth and the known sauropod taxa from the Aptian/Albian of North America, a reasonable working hypothesis is that this tooth belongs to a titanosauriform, likely *Sauroposeidon*.

THEROPODA Marsh, 1881

CARNOSAURIA von Huene, 1920

ALLOSAUROIDEA Currie & Zhao, 1993

ALLOSAURIDAE Marsh, 1878

*Acrocanthosaurus atokensis* Stovall & Langston, 1950

**Referred material.** UA-2016-13-039 to UA-2016-13-040, UA-2016-13-043 to UA-2016-13-045, UA-2016-13-058, UA-2016-13-77 to UA-2016-13-79; UA-2016-13-083, UA-2016-13-084.

**Figure 17:**
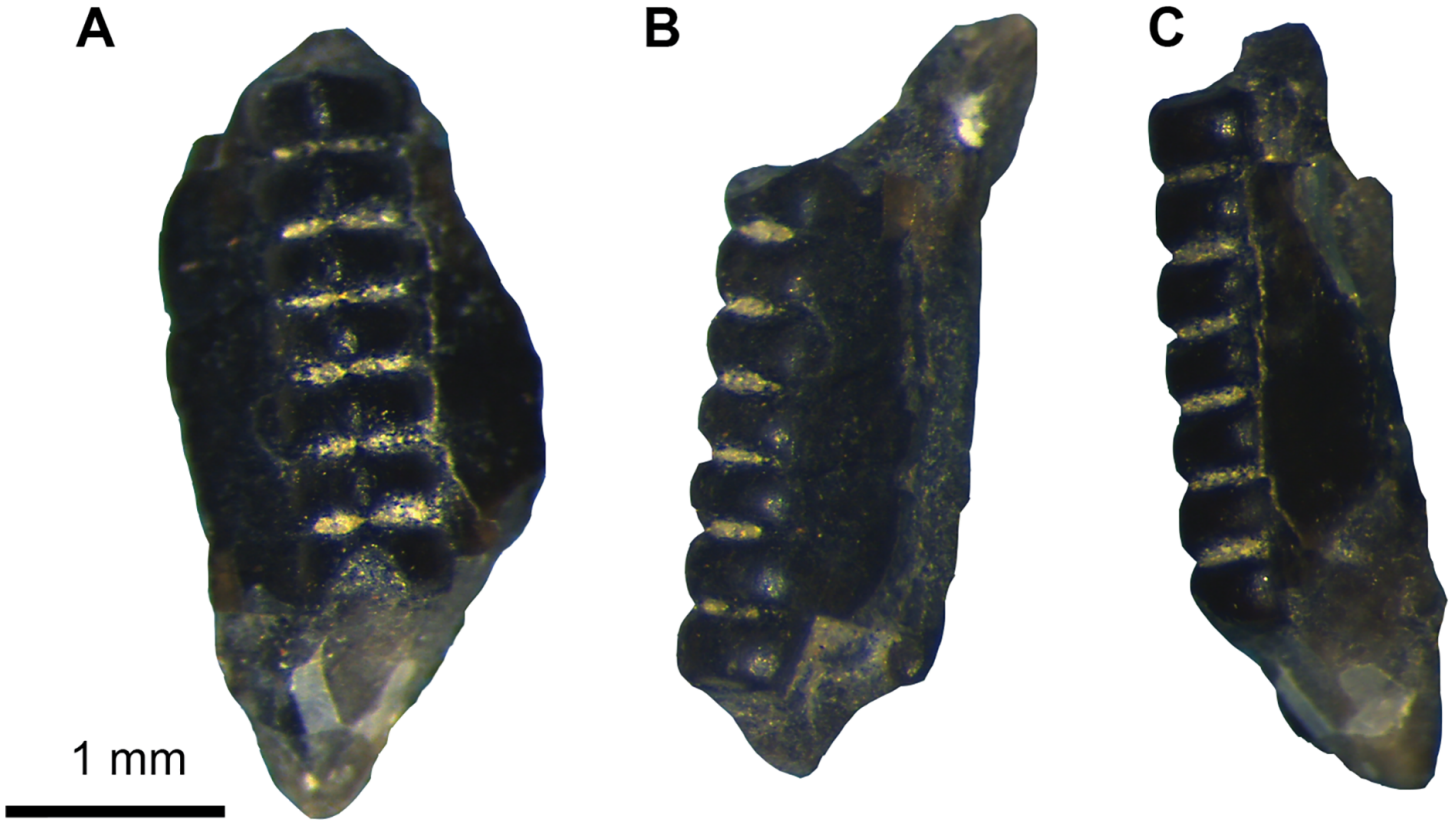
cf. *Acrocanthosaurus*, denticular tooth fragment. UA-2016-13-83 in (A) dorsal and (B–C) lateral views. Scale bar = one cm.

**Figure 18:**
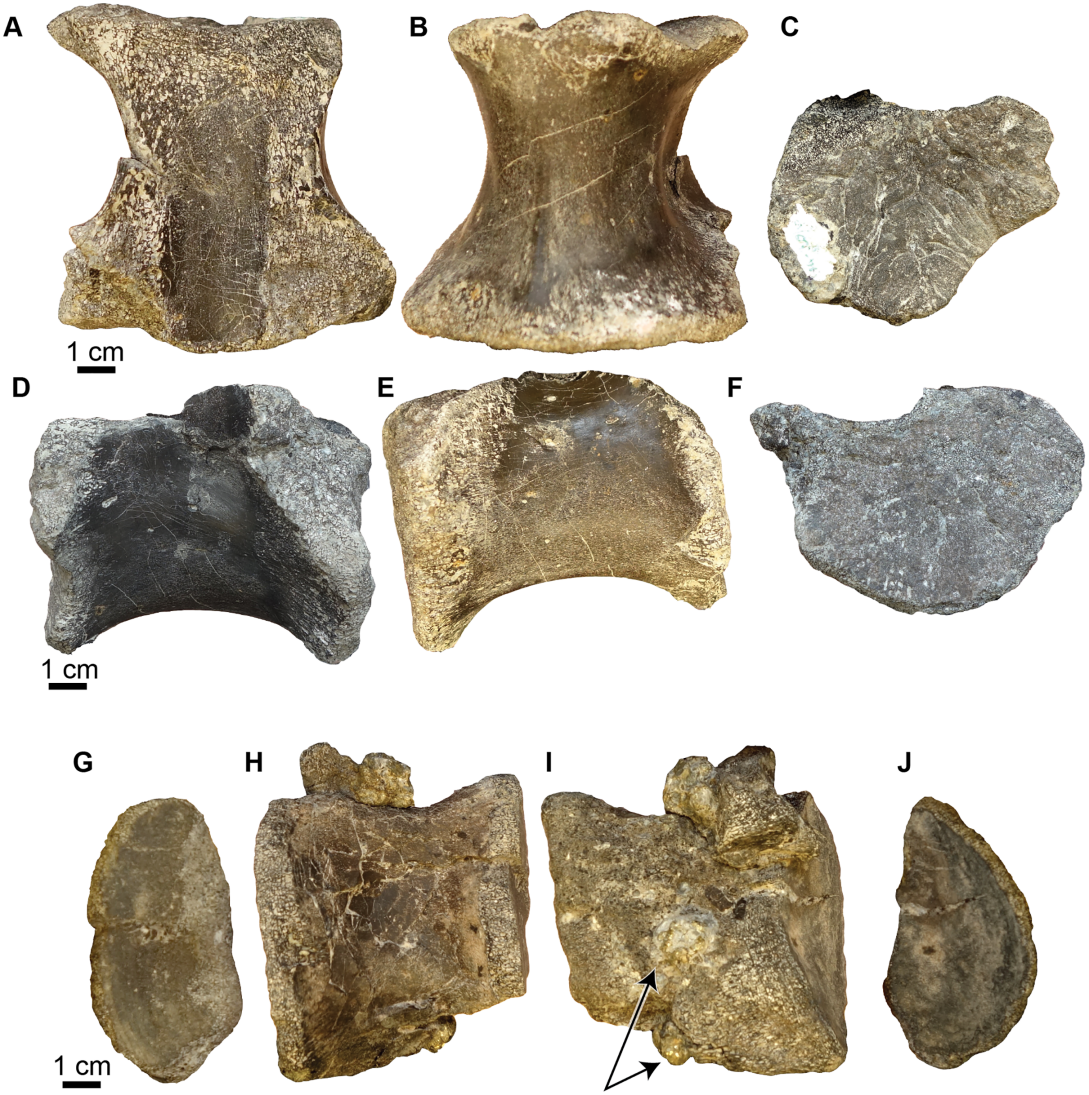
*Acrocanthosaurus* vertebrae. (A–F) Sacral vertebra (UA-2016-13-40) in dorsal (A), ventral (B), anterior (C), left lateral (D) right lateral (E), and posterior (F) views. The rugose nature of the anterior and posterior surface of the centrum suggests this specimen is from a juvenile. Scale bar = one cm. (G–J) A partial dorsal vertebra (UA-2016-13-39) in anterior (G), right lateral (H), left lateral (I), and posterior (J) views. Arrows point to clusters of pyrite. Scale bars = one cm.

**Figure 19:**
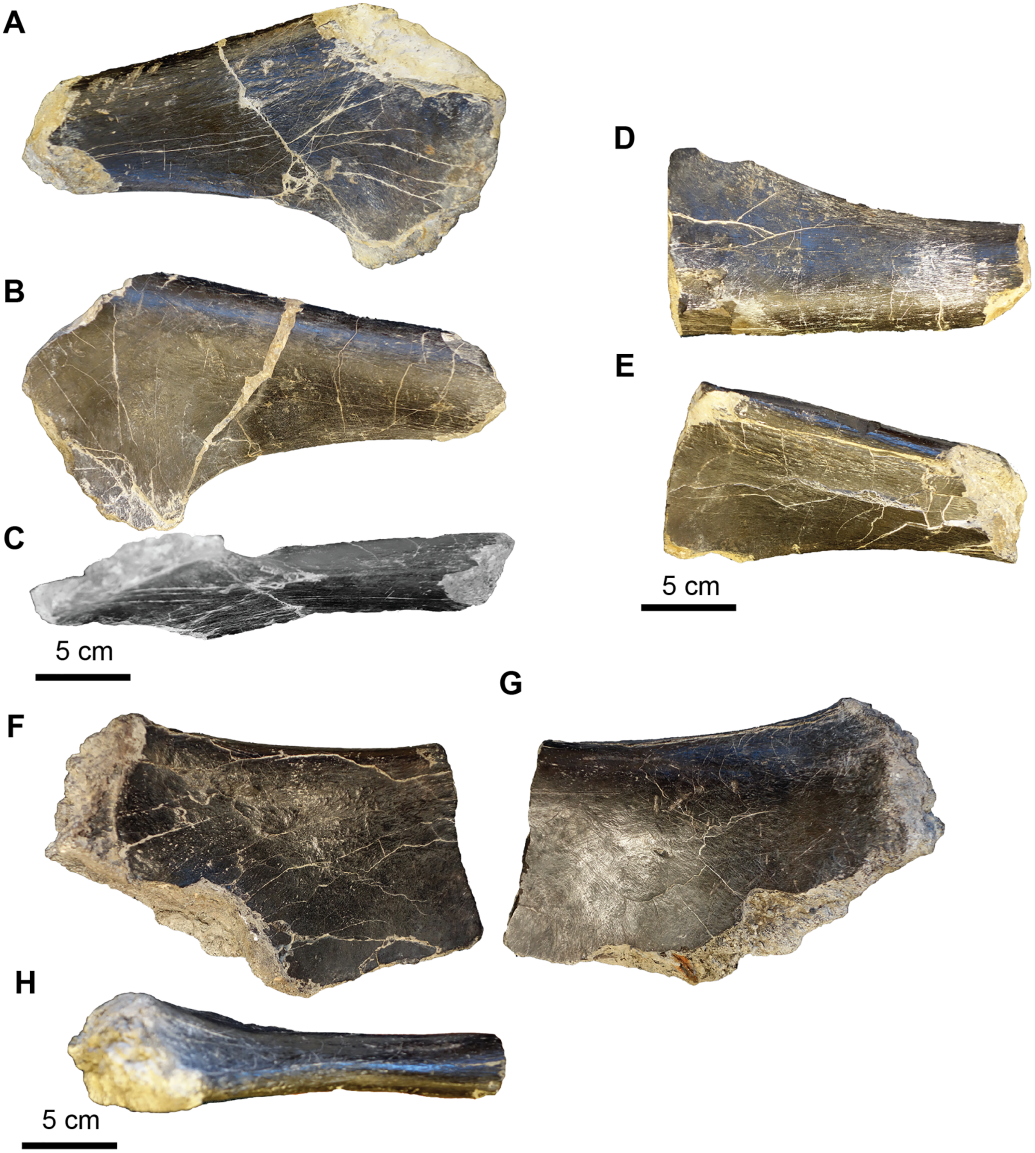
*Acrocanthosaurus* hip elements. (A–C) Proximal end of the left pubis (UA-2016-13-77) in lateral (A), medial (B), and ventral (C) views. (D–E) Proximal end of right pubis (UA-2016-13-78) in medial (D) and lateral (E) views. (F–G) Proximal ischium? (UA-2016-13-79) in lateral (F–G) and dorsal (H) views. Scale bars = five cm.

**Figure 20:**
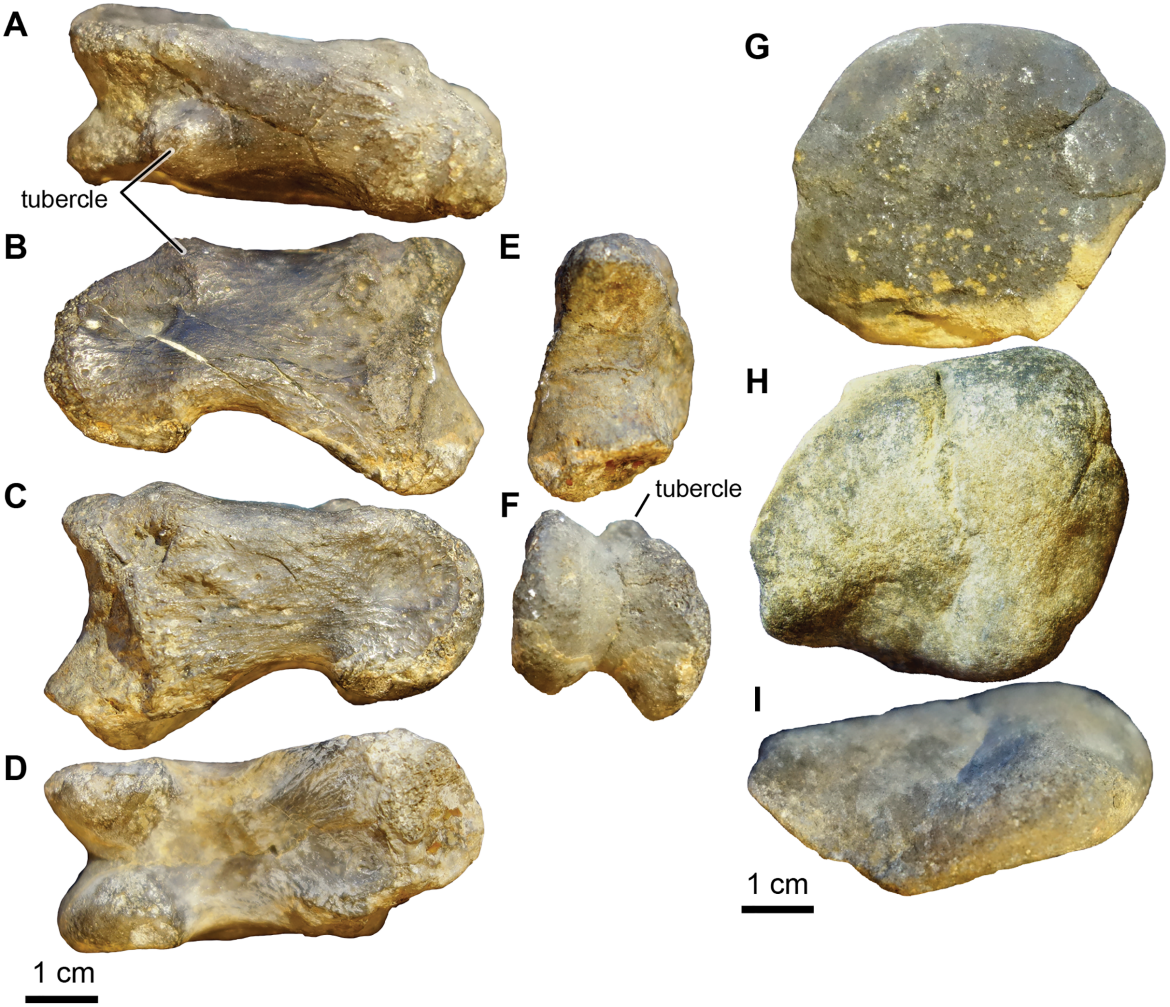
*Acrocanthosaurus* manus elements. (A–F) Phalanx 1 from right digit II (UA-2016-13-45) in dorsal (A), left lateral (B), right lateral (C), ventral (D), proximal (E), and distal (F) views. (G–I) Ulnare? (UA-2016-13-58) in distal (G), proximal (H), and lateral (I) views. Scale bars = one cm.

**Description and comments**. A tooth fragment bearing denticles (Fig. 17), two vertebrae (Fig. 18), right manus elements including phalanx I from right digit II and possibly an ulnare (Fig. 19), and both left and right proximal pubis and a proximal ischium (Fig. 20) from a juvenile large theropod were recovered from the site.

The tooth fragment (Fig. 17) contains approximately three denticles per mm. This is consistent with *Acrocanthosaurus*, in which Currie & Carpenter (2000) note that between 12.5 and 17.5 denticles are present per five mm. The Holly Creek Formation specimen would contain ~15 denticles per five mm.

The two vertebral elements include one sacral vertebra (Figs. 18A–18F) and one dorsal vertebra that is slightly crushed and sheared in half down the midline of the centrum (Figs. 18G–18J). Both vertebrae are amphicoelous. The dorsal vertebra is 7.2 cm long (anterior to posterior). The centrum is 7.6 cm measured from the ventral to dorsal end on the posterior articular surface and 7.2 cm measured from ventral to dorsal end on the anterior articular surface. The width of the centrum could not be measured since half of it is sheared off. The length of the vertebra is ~50% of the length of the vertebra of the adult holotype (dorsal vertebrae range between 15.3 and 10.7 cm). This element also has several nodules of cubic pyrite associated with it. The neural processes are crushed and mostly missing. The sacral vertebra is slightly longer than wide. We identify this element as a sacral vertebra based on the highly rugose nature of the articular surfaces, similar to the sacral vertebrae of *Allosaurus* figured in Madsen (1993). The posterior end is 8.8 cm wide and the anterior end is 7.7 cm wide. The vertebra is 9.2 cm long (anterior to posterior) and the narrowest point in the center of the centrum is 4.7 cm. There is a prominent keel running down the midline of the ventral surface of the vertebrae. The transverse processes are also missing. The rugose nature of the articular surface suggests it was not fused to its adjacent sacral vertebrae and is thus likely from a juvenile. No sacral vertebrae are described by Stovall & Langston (1950), Currie & Carpenter (2000), or D’Emic, Melstrom & Eddy (2012), so direct comparison with *Acrocanthosaurus* described by these authors is not possible. However, the fourteenth dorsal vertebra of the holotype specimen OMNH 10,146 is 12.5 cm long, 3.3 cm longer than the sacral vertebrae described here. Thus, the sacral vertebra is ~70% the size of the 14th dorsal vertebra from the holotype specimen.

The hip elements (Fig. 19) are tentatively identified as a proximal end of the left and right pubis and a possible proximal end of a left ischium. All three elements are broken on both distal and proximal ends, so it is not possible to estimate the lengths of the elements. The cross section of the left and right pubis is ovoid in shape. The long axis of the left pubis is 6.0 cm and the short axis is 4.0 cm. The long axis of the right pubis is 5.8 cm and the short axis is 3.6 cm.

The right phalanx 1 from digit II is 5.3 cm long (approximately half the size of the holotype from the Antlers Formation) (Figs. 20A–20F). It is 2.2 cm wide measured from the distal articular condyles and 2.0 cm wide at the narrowest part of the shaft. The intercondyle sulcus is 4 mm deep. The phalanx is 3.5 cm tall at the widest point at the proximal end of the phalanx and 2.0 cm tall at the narrowest point, just proximal to the distal condyles. There is a prominent tubercle, presumably for ligament attachment on the ventral surface of the medial condyle.

The collection includes a quadrangular, biscuit-like bone that is 5.4 cm wide at its widest point and 4.2 cm at its narrowest point (Figs. 20G–20I). It has a broad groove that results in a convex surface and a prominent broad ridge on either side of the groove. The element looks vaguely like the ulnare illustrated by Currie & Carpenter (2000, Figs. 10K–10L) in anterior-posterior view. It is roughly the same size as the holotype ulnare.

These elements are similar in shape to the only known large theropod from the Early Cretaceous of the southern US, *Acrocanthosaurus atokensis* (Stovall & Langston, 1950; Currie & Carpenter, 2000; D’Emic, Melstrom & Eddy, 2012). Comparison to the holotype specimen, OMNH 10146 from the Antlers Formation of Oklahoma, confirms that the Holly Creek specimens likely belong to one or more immature individuals, in that comparable elements (vertebrae and phalanx) are much smaller than those of the holotype specimen. The type specimen of *Acrocanthosaurus* was collected in eastern Oklahoma, close to the border with Arkansas, and very large theropod tracks attributed to *Acrocanthosaurus* sp. were located stratigraphically above (De Queen Formation) the vertebrate remains in the same site (Platt et al., 2018).

COELUROSAURIA von Huene, 1920

Family incertae sedis

cf. *Richardoestesia* Currie, Rigby & Sloan, 1990.

**Referred Material.** UA-2016-13-178 (one complete tooth).

**Figure 21:**
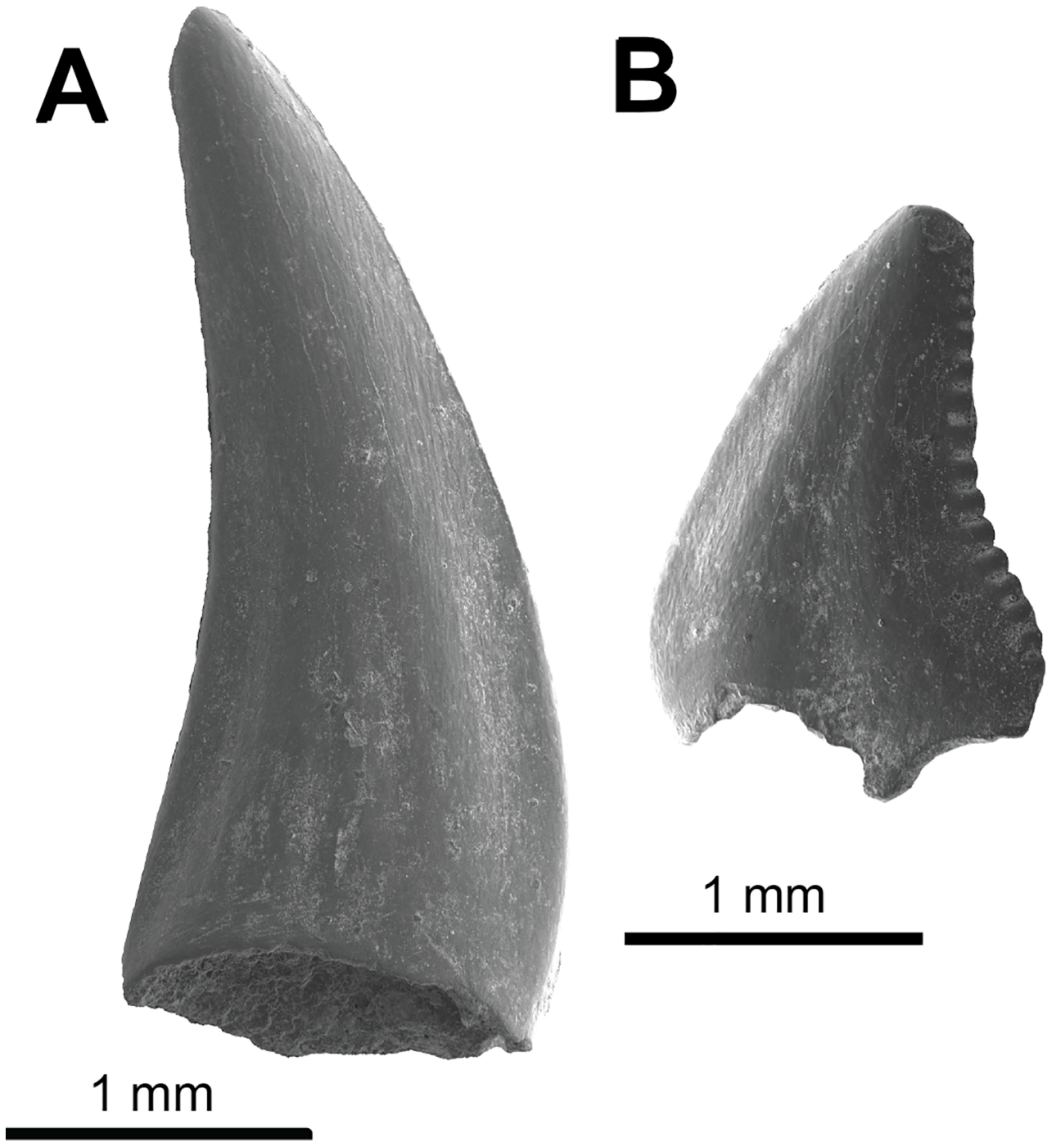
Coelurosauria teeth. (A) cf. *Richardoestesia* sp. (UA-2016-13-178) and (B) *Deinonychus antirrhopus* (UA-2016-13-177). Scale bars = one mm.

**Description and comments**. A small edenticulate and recurved tooth is 3.3 mm tall with well-developed carinae on both mesial and distal margins (Fig. 21A). These unusual characteristics are comparable to conditions seen in the genus *Richardoestesia*. Because this tooth lacks serrations, it may be confused with teeth from a pterosaur or bird; however, nearly identical teeth are also known from the uppermost Cedar Mountain Formation of Utah, some bearing small denticles on the posterior carina (Frederickson, Engel & Cifelli, 2018). *Richardoestesia* teeth are also known from the Aptian/Albian Cloverly Formation (Oreska, Carrano & Dzikiewicz, 2013), the Arundel Clay (Frederickson, Lipka & Cifelli, 2018), and specimens currently under study from the Antlers Formation of Oklahoma (OMNH 33321, 33513, 34031, 34122, 60836, 60985). Isolated teeth referable to *Richardoestesia* have been reported from many other rock units, spanning the Barremian–Maastrichtian (Kirkland, Lucas & Estep, 1998; Kirkland et al., 1999; Larson & Currie, 2013). Based on its elongate teeth, *Richardoestesia* is hypothesized to have been a piscivorous carnivore (Baszio, 1997), similar in ecology to modern wading birds (Frederickson, Engel & Cifelli, 2018).

MANIRAPTORA Gautier, 1986

DROMAEOSAURIDAE Matthew & Brown, 1922

*Deinonychus* Ostrom, 1969a

*Deinonychus antirrhopus* Ostrom, 1969a

**Referred Material.** UA-2016-13-081; 2016-13-082; UA-2016-13-179; 2016-13-177; four complete teeth.

**Figure 22:**
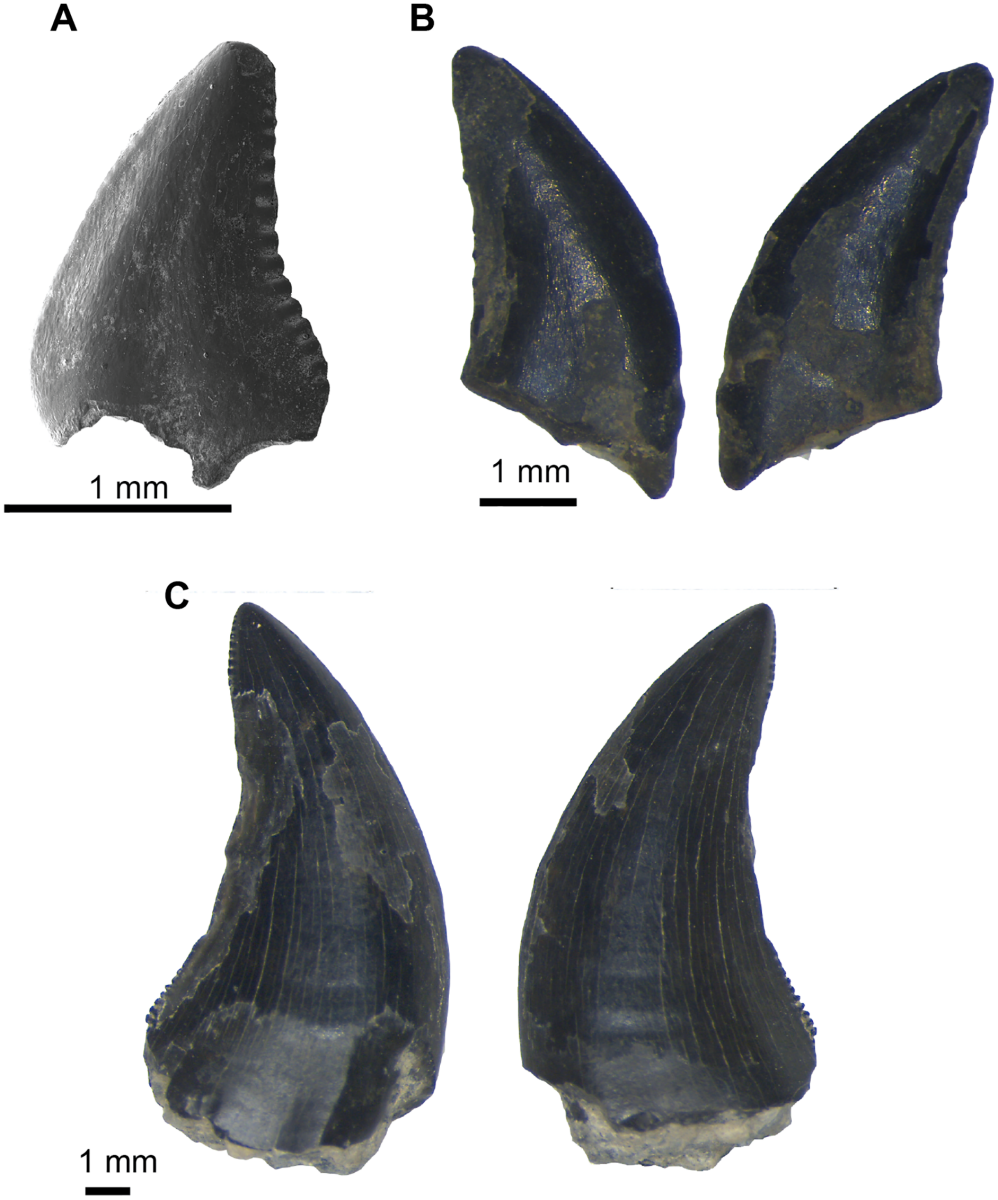
*Deinonychus* teeth: size variation. (A) Smallest tooth, from a presumed hatchling (UA-2016-13-177, also shown in Fig. 20B, 1.9 mm crown height). (B) Slightly larger specimen, also presumably from an immature individual (UA-2016-13-82, 4.7 mm crown height). (C) Largest *D*. *antirrhopus* tooth from the Briar Site locality, presumably belonging to a mature individual (UA-2016-13-81, 12.9 mm crown height).

**Description and comments**. We identify four small teeth (Figs. 21B, 22) from the Briar Site as belonging to *Deinonychus antirrhopus*. They range in crown height size from 1.9 mm to 12.9 mm. The teeth are recurved, with approximately 10 denticles per mm for the smallest tooth UA 2016-13-177, which is only 1.9 mm in height, likely from a juvenile or hatchling and consistent with the generally small size observed for similar teeth by Brinkman, Cifelli & Czaplewski (1998) and Frederickson, Lipka & Cifelli (2018) for *D. antirrhopus* teeth from the Antlers Formation locality OMNH V706 of Atoka County, Oklahoma. UA-2016-13-82 is also small, only 4.7 mm in height, and is likely from a juvenile. The denticles are poorly preserved in this specimen. UA-2016-13-81 is much larger, 12.9 mm, and is probably from an adult. It has approximately six denticles per mm. The denticles on the distal side are most prominent and less prominent on the mesial side. These features are most similar to *Deinonychus antirrhopus* (Ostrom, 1969b). First described on the basis of relatively complete cranial and postcranial remains from the Cloverly Formation of Montana (Ostrom, 1969a, 1969b; Maxwell & Ostrom, 1995), *Deinonychus antirrhopus* is also known from less complete yet diagnostic craniodental and skeletal fossils from the Antlers Formation of Oklahoma (Brinkman, Cifelli & Czaplewski, 1998). Our provisional referral of isolated teeth to the species, herein, is based on comparison with large samples of teeth, including substantial variation in size and presumed ontogenetic age, from both these units in which occurrence is unambiguously established. Like all other *D. antirrhopus* teeth, the distal denticles are large and angled in a slightly apical direction, while the mesial denticles are nearly imperceptible, which is likely a result of wear; not an uncommon occurrence in teeth from the Antlers Formation of Oklahoma (Brinkman, Cifelli & Czaplewski, 1998).

*Deinonychus* has been reported from other Cretaceous rock units across North America; we are able to comment only on those for which we have made firsthand observations on relevant specimens. Among these, the Arundel Clay is notable for its geographic location, on the US eastern seaboard. As with specimens from the Briar Site, isolated teeth from the Arundel are indistinguishable from the OMNH samples of *D*. *antirrhopus* from the Cloverly and Antlers formations (Lipka, 1998; Frederickson, Lipka & Cifelli, 2018). *Deinonychus* has also been reported from the upper part of the Cedar Mountain Formation, Utah: cranial and postcranial remains from the Ruby Ranch Member, reportedly in private hands and not yet described; and isolated teeth from the Mussentuchit Member (Carpenter et al., 2002). Detailed study of large samples of isolated teeth at the NCSM (Avrahami et al., 2018) and the OMNH (Cifelli et al., 1999b; Frederickson, Engel & Cifelli, 2018) have failed to positively identify *Deinonychus* from the Mussentuchit Member of the Cedar Mountain Formation. Although it is not specifically identified as *Deinonychus*, a single tooth from the Proctor Lake Dinosaur Locality in the Twin Mountains Formation of Texas is attributed to Dromaeosaurinae (in which *Deinonychus* is sometimes placed). This specimen also has a distinctly larger serrated carina on the distal side of the tooth compared to that on the mesial surface (Winkler et al., 1988; Adams, 2019).

ORNITHISCHIA Seeley, 1887

THYREOPHORA Nopcsa, 1915

ANKYLOSAURIA Osborn, 1923

NODOSAURIDAE Marsh, 1890

Genus and species indet.

**Referred material:** UA-2016-13-049 to UA-2016-13-057.

**Figure 23:**
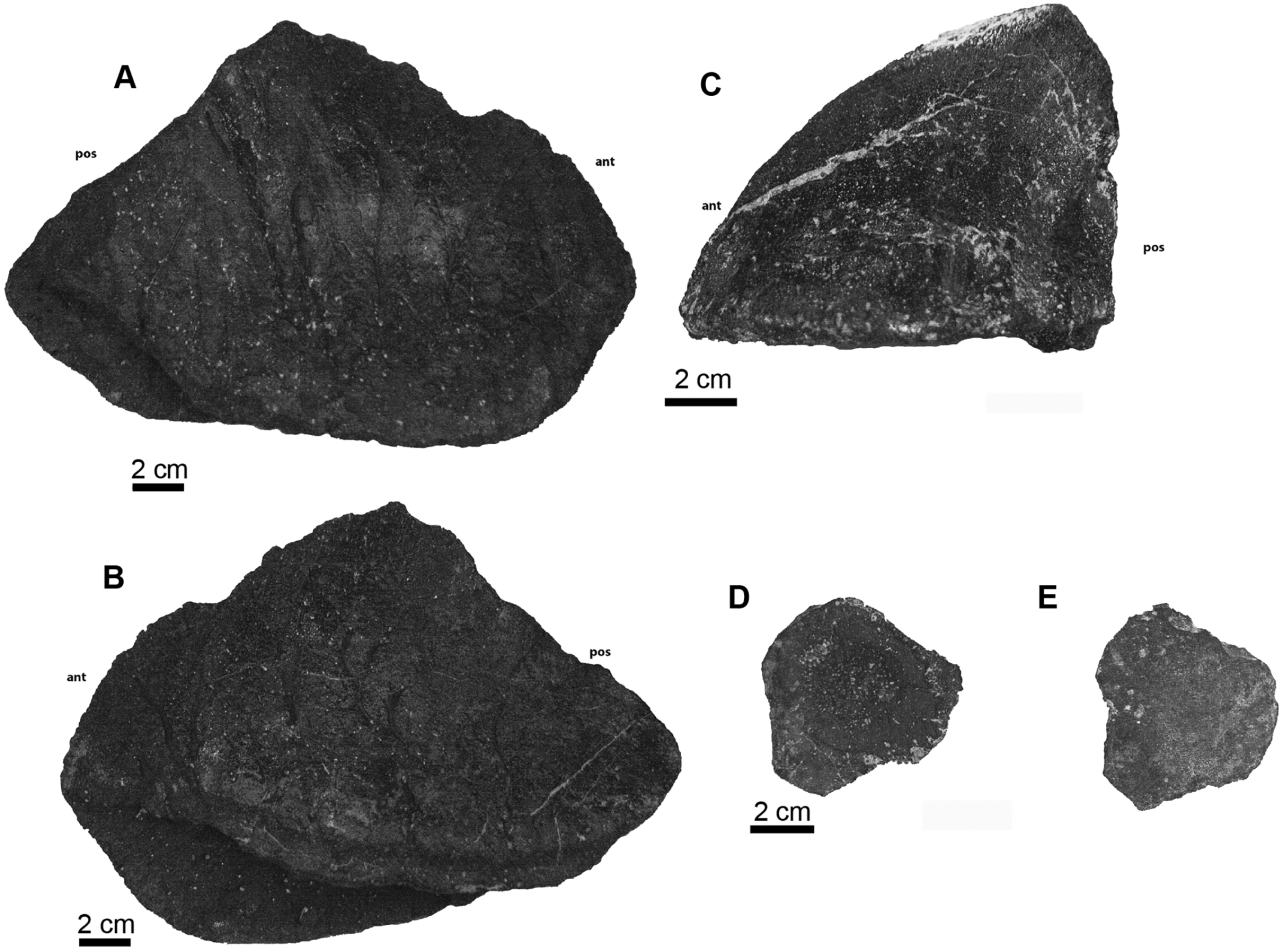
Nodosauridae (Ankylosauria) dermal armor. (A–B) Right caudal plate (UA-2016-13-49) in ventral (A) and dorsal (B) views. (C) A dermal spine (UA-2016-13-50) in lateral view. (D–E) A dermal scute (UA-2016-13-51) in dorsal (D) and ventral (E) views.

**Description and comment**. Ankylosaur material recovered from the Briar locality consists of two small osteoderms, a small spine, and a caudal plate (Fig. 23). Osteoderms (Figs. 23D, 23E) are sub-rounded and 5.8 cm across. The external surfaces are flat around the edges and build to a small oval rise approximately 0.5 cm tall. The internal surface displays a characteristic crosshatched pattern. The spine (Fig. 23C) is small and sub-triangular, posteriorly sloping to almost a straight edge, with a height of 12.4 cm. The base is concave and sub-ovoid in shape. It is 12 cm long and 7.8 cm at its widest point, tapering to 3.8 cm at the posterior edge. The plate (Figs. 23A, 23B) is a right caudal with a concave, elongate, ovoid base. The base is 5.6 cm at its widest point at the center, narrowing to two cm at either end. The ventral edge of the base is extended out 4.5 cm on the anterior side. The dorsal side extends posteriorly but does not overlap the ventral surface. The dorsal surface is 16 cm at its tallest, the ventral surface is slightly larger at 17.8 cm. Both are 24.6 cm in length. It displays well-defined grooves, which in life presumably held blood vessels, on both sides. There are possible long and conical tooth marks extending toward the center from both proximal and distal lateral edges on the ventral face. The posterior part of the plate is incomplete, though likely not more than ~2 cm is missing.

We refer these specimens to Nodosauridae indet. Geographical location would suggest links to ankylosaurs of the Pawpaw Formation, such as *Pawpawsaurus* Lee, 1996 and *Texasetes* Coombs, 1995. The Holly Creek Formation is similar in age to the Cloverly, which contains the nodosaurid *Sauropelta* Ostrom, 1970, and part of the Cedar Mountain Formation, which holds a diversity of ankylosaur material such as *Animantarx* Carpenter et al., 1999, *Cedarpelta* Carpenter et al., 2001, and *Peloroplites* Carpenter et al., 2008 in strata that are described as the somewhat younger Mussentuchit Member.

Although polacanthid ankylosaurs such as *Gastonia* Kirkland, 1998 are found in the Cedar Mountain Formation, they occur in the lower members, predominantly the Yellow Cat, which is Barremian or older in age, and in the Berriasian-Valanginian Lakota Formation of South Dakota (Pereda-Suberbiola, 1994; see age estimate by Sames, Cifelli & Schudack, 2010; Cifelli, Davis & Sames, 2014). Therefore, the chance of material belonging to a polacanthid ankylosaur is more unlikely as none is yet known from beds younger than Barremian in age.

MAMMALIA Linnaeus, 1758

**Figure 24:**
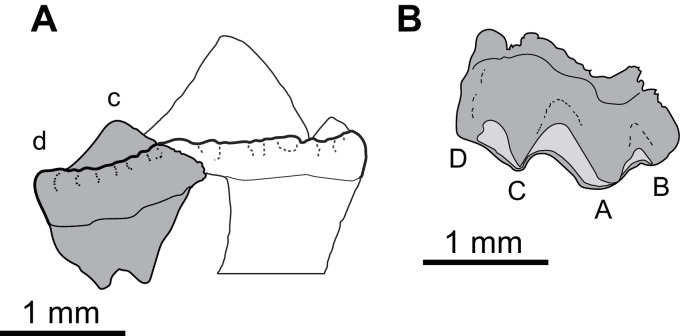
Mammalia. (A) Alticonodontinae indet. (Eutriconodonta: Triconodontidae), fragment of left p2 in lingual view. Restored outline (anterior part, to the right) based on cf. *Astroconodon* sp., MCZ 20023. (B) Spalacolestinae indet. (Trechnotheria: Spalacotheriidae), left P2? in lingual view. Letters denote cusps mentioned in the text; terminology from Crompton & Jenkins Jr. (1968); scale bar = one mm. (Original specimens mislaid; drawings made from photographs.)

**Comment**. Screen-washing of rock matrix from the Briar Site locality yielded two partial mammal teeth (Pittman & Bell, 2002). Unfortunately, these have been mislaid and cannot be located at present. We have several photographs that, although of poor quality, reveal enough detail for identification at the family-group level, brief description, and outline drawings (Fig. 24). Measurements are based on the scale bars included in the photos.

EUTRICONODONTA Kermack, Mussett & Rigney, 1973

TRICONODONTIDAE Marsh, 1887

ALTICONODONTINAE Fox, 1976

Genus and species indet.

**Description and comments.** One of the mammal specimens represents the distal part of a triconodontid anterior premolar, probably a left p2 (Fig. 24A), preserving cusps c and d, the lingual cingulum, and part of the distal root. It can be distinguished as an alticonodontine, rather than a triconodontine, based on the low height of cusp c and on characteristics of the underlying distal root, which is anteroposteriorly elongate, laterally compressed, and somewhat distally oriented. The strong development of the lingual cingulum suggests that the premolar pertains to the lower dentition. Cusp d is expressed as the raised distal tip of the cingulum and is sharply pointed. As preserved, the specimen is about 1.4 mm long. Comparison to p2 of *Astroconodon denisoni* Patterson, 1951; (see Turnbull & Cifelli, 1999), *Corviconodon utahensis* Cifelli & Madsen, 1998, and an unnamed species currently under study from the Cloverly Formation of Montana (MCZ 19969, 19974, 200023) yields an estimated total length of 2.74 mm for the Holly Creek fossil, which is broadly similar to P2/p2 in the comparator taxa.

TRECHNOTHERIA McKenna, 1975

SPALACOTHERIIDAE Marsh, 1887

SPALACOLESTINAE Cifelli & Madsen, 1999

Genus and species indet.

**Description and comments.** The other mammal specimen from the Briar Site locality is a nearly complete, highly distinctive premolar (Fig. 24B). The tooth bears three main cusps, A–C; the crest ascending distally from cusp C terminates sharply at the distal margin of the crown, which appears to have had a weakly distinct cusp D when unworn. The tooth is broken just mesial to cusp B. Cusp A is the tallest of the three main cusps; cusp B is slightly taller than cusp C and, of the two, is placed noticeably closer to cusp A (in these respects differing from lower premolars of spalacolestines; Cifelli, 1999). The tooth shows considerable wear, and a strap-like surface of exposed dentin extends the length of the tooth crown, running between the cusp apices and disto-superiorly from the apex of cusp C. Wear appears to have been strongest on the distal surface of cusp A, at the mesial and superior parts of the notch separating that cusp from cusp C. The base of the crown displays a prominent ventral flexure between the positions of the two roots (which are almost completely broken away, except for two small stubs representing the base of the distal root), directly above cusp A.

Based on cusp proportions and spacing, we identify this partial tooth as the penultimate upper premolar of a spalacolestine “symmetrodontan” (*sensu* Kielan-Jaworowska, Cifelli & Luo, 2004). The only spalacolestine for which a complete upper cheektooth series is known is *Lactodens sheni* Han & Meng, 2016; an unnamed species from the Cloverly Formation of Montana is known by a complete lower cheektooth series (Cifelli et al., 2000). *Lactodens* has five lower premolars (as does the Cloverly taxon) but only three in the upper dentition; on this basis we provisionally identify the Holly Creek specimen as a left P2?. Comparison with *Lactodens* suggests that the preserved anteroposterior length of this P2?, 1.79 mm, closely approximates the full length of the specimen.

## Discussion

**Comparison to other Early Cretaceous faunas.** The Early Cretaceous has been plagued by a scarcity of accurate, detailed, and consistent chronologic studies. Some of this stems from our inability to date original ash layers, the fact that many Lower Cretaceous formations include multiple, major unconformities that are difficult to distinguish in the rock record, and confusion as to stage nomenclature. Nonetheless, assemblages from at least four units can be compared to the Holly Creek fauna and one of these (the Trinity Group and its correlative Antlers fauna) can be directly correlated lithologically. The other three include assemblages from the Cloverly, Cedar Mountain, and Arundel formations of Montana/Wyoming, Utah, and Maryland, respectively.

**Figure 25:**
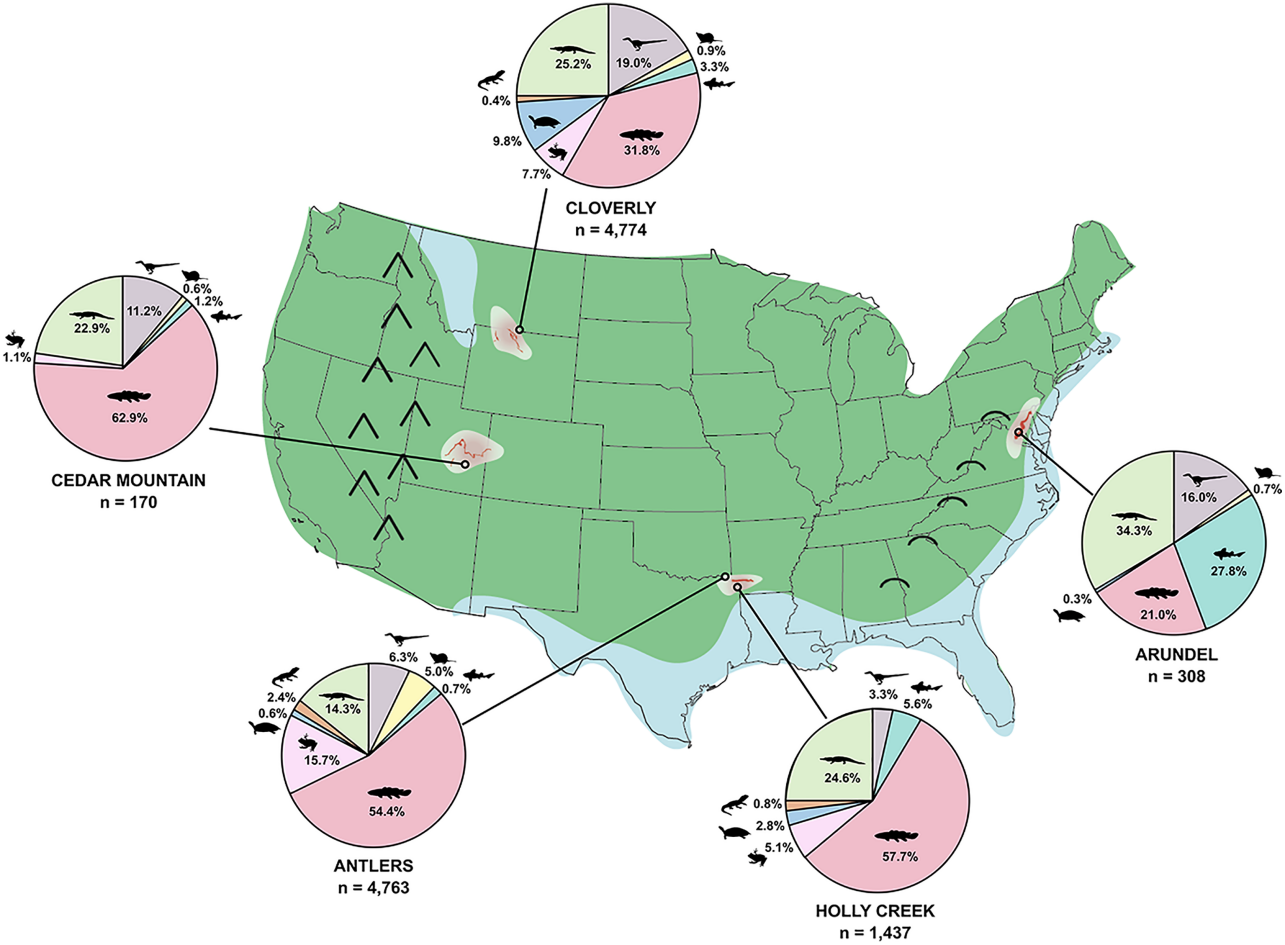
Faunal comparison. A comparison of faunal assemblages from medial Cretaceous formations of North America (based on Frederickson, Lipka & Cifelli, 2018). Data from: OMNH collections Antlers and Cedar Mountain (Ruby Ranch Member, Hotel Mesa V857) formations. Cloverly Fm. from Oreska, Carrano & Dzikiewicz (2013) and Arundel Fm. from Frederickson, Lipka & Cifelli (2018). Paleogeographic map for the Late Aptian-Early Albian. Green represents land, blue represents shallow seas. Highlands in the west represent the Sevier Mountains and, in the east represent the Appalachian “hills.” Outcrop distribution of the four formations are outlined in dark red, superimposed on a rough extent of the paleo-depositional basin (transparent pale pink).

The Antlers Formation of Oklahoma and north central Texas is considered correlative to the combined Twin Mountains, Glen Rose, and Paluxy formations of the Trinity Group as exposed in central Texas (Jacobs & Winkler, 1998). The vertebrate fauna described herein is very similar to that of the Trinity Group/Antlers Formation (Fig. 25). In overall composition, both are characterized by abundant fossils of bony fishes and crocodilians. Like the Antlers fauna, it contains paramacellodid-cordylid grade lizards, teeth of coelognathosuchian crocodyliforms, the large allosauroid *Acrocanthosaurus atokensis, Sauroposeidon*, the large solemydid turtle *Naomichelys speciosa*, and both triconodontid and spalacotheriid mammals (Langston, 1974; Cifelli et al., 1997a; Jacobs & Winkler, 1998; Nydam & Cifelli, 2002; D’Emic, Melstrom & Eddy, 2012; D’Emic, 2013). Similarly, the Twin Mountains Formation contains the neosuchian crocodyliform *Paluxysuchus* (Adams, 2013). Unlike the Trinity Group and the Antlers, no evidence of the ornithopods *Tenontosaurus* Ostrom, 1970 or *Convolosaurus* Andrzejewski, Winkler & Jacobs, 2019 are present either as trace fossils or as body fossils in the Holly Creek or De Queen Formation. Absence of *Tenontosaurus* is particularly striking in view of its considerable abundance in the Antlers Formation of Oklahoma (Brinkman, Cifelli & Czaplewski, 1998). Another notable difference is the presence, in the Holly Creek fauna, of a nodosaurid ankylosaur. This is a first occurrence in the Trinity Group. While absence data must be interpreted cautiously, it is worth pointing out that ankylosaur fossils are easily recognized as such, even if highly fragmentary, and that reasonable samples of the macro-and microfauna are known from both Oklahoma and Texas, but as of yet, none are from ankylosaurs. The geographically closest known Early Cretaceous ankylosaurs are *Silvisaurus* from the Dakota Formation of Kansas (Eaton, 1960) and the possibly synonymous *Texasetes* (Coombs, 1995) and *Pawpawsaurus* from the Paw Paw Formation (Albian, Washita Group) of Texas in Tarrant County (Lee, 1996). These occurrences are stratigraphically higher than the Holly Creek Formation. The caudal and dorsal plates of the Holly Creek nodosaur are similar to those referred to the nodosaurid *Sauropelta* from the Cedar Mountain Formation of Utah and Cloverly Formation of Wyoming.

Like the fauna of the Cedar Mountain Formation, the Holly Creek fauna also preserves *Acrocanthosaurus*, titanosauriform sauropods, *Naomichelys speciosa*, pycnodont fish, coelognathosuchian crocodyliforms, and mammals belonging to Triconodontidae and Spalacotheriidae. Kirkland et al. (2016) suggest the Cedar Mountain Formation preserves six faunal assemblages at the genus and species level and three major, paleogeographically significant faunal assemblages based on differences at higher taxonomic divisions. The three main assemblages include a lower “polacanthid fauna” that includes polacanthid ankylosaurs, spatulate-toothed sauropods, basal styracostern iguanodontids, and large dromaeosaurids (*Utahraptor*). The medial fauna, termed the “tenontosaurid” fauna, is poorly known. It includes one or more member each of nodosaurid ankylosaur, slender-toothed titanosauriform sauropod, a basal iguanodontian like *Tenontosaurus*, and an allosauroid theropod similar to *Acrocanthosaurus*. Most of this fauna is preserved within the Ruby Ranch Member of the Cedar Mountain Formation. The upper *Eolambia* fauna includes hadrosauroid igunodontians as well as marsupials, oviraptorids, a neoceratopsian, and tyrannosauroid dinosaurs that evolved in Asia, suggesting a connection between North America and Asia through an Alaskan land bridge (Cifelli et al., 1997b). This uppermost fauna contains abundant, well-represented lizard and mammal assemblages, including both triconodontids and spalacotheriids (Cifelli & Madsen, 1998, 1999). The Holly Creek fauna is most similar to the assemblage from the Ruby Ranch Member. Recent chronostratigraphic work by Ludvigson et al. (2010, 2015) and Montgomery (2014) suggest a late Aptian to Albian age for the Ruby Ranch Member, consistent with biostratigraphy of the Trinity Group in Arkansas. *Acrocanthosaurus*, nodosaurid ankylosaurs (including one with armor like *Sauropelta*), and slender-toothed titanosaurs (*Sauroposeidon*) found within the Holly Creek fauna of this study are similar to the “medial” Cedar Mountain fauna. The brachiosaurid *Abydosaurus mcintoshi* was described as hailing from the Mussentuchit Member (Chure et al., 2010), but more recent study suggests that it may have come from strata correlative to the Ruby Ranch Member (Kirkland et al., 2016). Maniraptoran theropods have not yet been reported from the Ruby Ranch Member; teeth similar to those of *Deinonychus* have been found in the Cenomanian Mussentuchit Member of the Cedar Mountain Formation (see comments in preceding descriptive section), however, the Mussentuchit fauna is likely younger than that of the Holly Creek Formation, with recent maximum depositional U/Pb detrital zircon ages of the upper Mussentuchit Member of 99.72 ± 0.12/0.12/0.16 MYA (Tucker et al., 2020). While refined age-constraints for parts of the Cedar Mountain Formation and the Holly Creek Formation hinder our ability to finely correlate these Early Cretaceous units, an Aptian-Albian relationship is evident, as is a link to the Ruby Ranch Member of the Cedar Mountain Formation.

Like the Cedar Mountain, there are occurrences of paramacellodid-cordylid grade lizards, *Acrocanthosaurus*, *Sauroposeidon*, *Sauropelta*, *Deinonychus*, and both triconodontid and spalacotheriid mammals from the Cloverly Formation, suggesting correlation to the fauna of the Cloverly. In a timely study by D’Emic et al. (2017), D’Emic et al. (2019) that re-evaluates the age of the Cloverly Formation of Wyoming, detrital zircon geochronology suggests that the Cloverly spans a much wider range in age, possibly Barremian to early Cenomanian rather than Aptian-Albian, contains many pervasive unconformities, and may have unrecognized, as yet to be identified taxonomic diversity similar to that of the Cedar Mountain Formation of Utah and the Trinity Group. Their study, however, suggests most vertebrates of the Cloverly are from the Little Sheep Mudstone and few taxa come from the lower parts of the Cloverly Formation. The Little Sheep Mudstone may span as much as 15 million years, from the late Aptian through much of the Albian. The actual stratigraphic range of various faunal elements remains to be reported. Nonetheless, general similarity of the Cloverly fauna to that of the Holly Creek Formation supports an Aptian–Albian age for the latter.

The Arundel Formation of the eastern seaboard contains a dinosaurian fauna similar to that of the Holly Creek Formation. Both contain an apex predator tentatively identified as *Acrocanthosaurus atokensis*, isolated maniraptoran teeth attributed to *Richardoestesia* sp. and *Deinonychus antirrhopus*, at least one ankylosaur, sauropod teeth (of *Astrodon* pattern) and postcranial material, and a triconodontid (Lipka, 1998; Cifelli et al., 1999a; Brownstein, 2017; Frederickson, Lipka & Cifelli, 2018; Hunt & Quinn, 2018). Conversely, the Holly Creek fauna contains different chondrichthyans and a much more abundant herpetofauna than the Arundel (Frederickson, Lipka & Cifelli, 2018), which itself is remarkable in its diversity and abundance of chondrichthyan fossils. The lack of *Tenontosaurus tilletti* (or similar basal iguanodontian) from the Holly Creek material is mirrored in the Arundel Formation, where ornithopod remains are rare (Galton & Jensen, 1979) compared to their abundance in the Antlers and Cloverly formations. The close proximity of the Holly Creek to the Antlers suggests the possibility that ornithopods were present in the area and that the absence of identified fossils may be linked to environmental preference in these herbivores, however, given the low sample size of Arkansas dinosaurs, we cannot discount a sampling bias.

**Paleobiogeography of the Early Cretaceous of North America.** The correlation of taxa from the Holly Creek, Cedar Mountain, Twin Mountains, Cloverly, Arundel and Antlers formations suggest a very wide geographic range of terrestrial vertebrates during the Aptian–Albian time period; in the case of certain well-preserved fossils, species-level identity exists between the Cloverly and Antlers assemblages (*e.g*., Brinkman, Cifelli & Czaplewski (1998); Nydam & Cifelli (2002); Cifelli & Davis (2015)). This is consistent with the observations of many Early Cretaceous researchers (Ostrom, 1970; Brinkman, Cifelli & Czaplewski, 1998; Jacobs & Winkler, 1998; D’Emic, Melstrom & Eddy, 2012) suggesting the presence of a geographically widespread but taxonomically depauperate fauna that spanned more than 15° paleolatitude and 30° paleolongitude. This contrasts with the highly diverse Campanian vertebrate faunas, including dinosaurs, squamates, and mammals, which show a high degree of provincialism (Cifelli, 1990, 1994; Rowe et al., 1992; Lehman, 2001; Gates et al., 2010; Carr et al., 2011; Nydam, Rowe & Cifelli, 2013; Nydam, 2013a, 2013b; DeMar, 2016). The marked rise in species richness during the Campanian suggests that, following the faunal turnover at the end of the Jurassic, prior to incursion of the Western Interior Seaway (WIS), and further tectonic influences of the Sevier Mountains, the faunal diversity was low. Following the evolutionary pressure (vicariance) caused by the rising WIS and Sevier Mountains and coincidental radiations in terrestrial vertebrates (*e.g*., mammals; Cifelli et al., 1997a; Cifelli, 2004; Wilson et al., 2012; Grossnickle & Newham, 2016), diversity greatly increased. Subsequent regression of the WIS and geodispersal resulted in a decrease in diversity in the late Maastrichtian (Erickson et al., 2004; Sampson & Loewen, 2005; D’Emic, Melstrom & Eddy, 2012). Changing local climatic parameters likely also controlled the environment and diversity and distribution of vertebrates. For example, Andrzejewski & Tabor (2020) found that the transition between the Aptian–Albian to Cenomanian is coincident with a transition from a cool dry climate to a wet and warm climate in the Cenomanian.

## Conclusions

We present the first description of a taxonomically diverse continental vertebrate fauna from the Early Cretaceous (Aptian–Albian) of Arkansas. This fauna was preserved in the ancient coastal plain of Arkansas and is dominated by semi-aquatic/aquatic taxa. The assemblage is similar to other Aptian–Albian faunas throughout North America, supporting the concept of a widespread but low-diversity Early Cretaceous fauna that spans from Montana to Arkansas, and as far east as Maryland. We present a new species of pycnodont fish, *Anomoeodus caddoi*, a likely relative of *Texasensis*, that existed in the waters of the ancient coastal plain; and the new lizard *Sciroseps pawhuskai*, belonging to the paramacellodid-cordylid grade that dominated herpetofaunas of the North American Aptian–Albian, represented by one of the most complete mandibles known from the North American Early Cretaceous. The addition of this faunal data set shows that nodosaurid ankylosaurs ranged as far south as Arkansas to the ancient Gulf Coast coastal plain. Additional high-precision dating of Early Cretaceous strata throughout the Western Interior Basin and the ancient Cretaceous Gulf coast is needed to understand more fully the roles that immigration events, tectonism, and global climate change may have played in the transition from Early to Late Cretaceous fauna.

This new fauna from Arkansas helps fill gaps in knowledge of poorly-known and understudied midwestern and southern faunas and offers a link between the well-known western (Laramidia) faunas and well-known eastern faunas (Appalachia). The material described here offers an exciting opportunity to understand the spatial links between western and eastern faunas of the late Early Cretaceous and temporal transition between the late Early Cretaceous to the Late Cretaceous. The material here described will offer plentiful material for further taxonomic, paleobiogeographic, and isotopic studies in the years to come.

## Supplemental Information

10.7717/peerj.12242/supp-1 Supplemental Information 1 Full specimen list. All specimens catalogued at the University of Arkansas Museum

10.7717/peerj.12242/supp-2 Supplemental Information 2 UA-2016-13-321, centrum; UA-2016-13-322, left neural arch. Turntable video showing dorsal crocodile vertebra in original specimen form as a 3D model from photogrammetry and 3D modeling software (UA-2016-13-322, centrum; UA-2016-13-323, left neural arch; UA-2016-13-324, right neural arch). Abbreviations: azo, prezygapophysis; pzo, postzygapophysis. Scale bar is six cm, which is the length of the centrum. There was significant distortion and displacement of elements during burial. The right postzygapophysis, along with the side of the neural process, was broken and turned downward to become vertically oriented. The left postzygapophysis was broken from its neural process and became affixed to the top of the right neural process. Compare this video to Video S2, which shows a reconstruction of this vertebra.

10.7717/peerj.12242/supp-3 Supplemental Information 3 UA-2016-13-321, centrum; UA-2016-13-322, left neural arch; UA-2016-13-323, right neural arch, reassembled. Turntable video showing the same vertebra in Video S1, here shown after 3D software was used to select parts of the 3D model that could be shifted and rotated so that broken edges matched, to attain life position of distorted parts. Same abbreviations and scale bar as in Video S1. Green colored parts of the 3D model show areas that occupy regions of stretching or voids after shifting and rotating of the right postzygapophysis was done; the green areas are akin to plaster filler areas in a real-world reconstruction. The left postzygapophysis was simply plucked off the top and placed on the left neural process so that it matched the orientation of the reconstructed right postzygapophysis (Compare to Video S1).

## Institutional Abbreviations

FUB: Freie Universität, Berlin, Germany
MCZ: Museum of Comparative Zoology, Cambridge, Massachusetts, USA
MNA: Museum of Northern Arizona, Flagstaff, Arizona, USA
NCSM: North Carolina Museum of Natural Sciences, Raleigh, North Carolina, USA
NHMUK: Natural History Museum, London, UK
OMNH: Oklahoma Museum of Natural History, Norman, Oklahoma, USA
SMP-SMU, SMU: Southern Methodist University, Shuler Museum of Paleontology, Dallas, Texas, USA
UA: University of Arkansas Museum, Fayetteville, Arkansas, USA
TMM: Texas Memorial Museum, Austin, Texas, USA

## Additional Information and Declarations

### Data Availability

The following information was supplied regarding data availability: The specimen list is available in the Supplementary File. All specimens are housed at the University of Arkansas Museum (https://fulbright.uark.edu/university-museum/). The exceptions, samples UA-2016-13-85 to UA-2016-13-293 and samples UA-2016-13-295-UA-2016-13-321, are temporarily housed for study at the Sam Noble Museum of Natural History at the University of Oklahoma and will be returned to the University of Arkansas Museum before the end of 2021. Sample UA-2016-13-294 is temporarily housed at Midwestern University and is in the process of being transferred back to the University of Arkansas Museum. A 3D model of one of the crocodiliform vertebra and attached neural processes is also available in the Supplementary Files as well as at MorphoSource. -Video 1: DOI 10.17602/M2/M369562. -Video 2: DOI 10.17602/M2/M369565.

### New Species Registration

The following information was supplied regarding the registration of a newly described species: Publication LSID: urn:lsid:zoobank.org:pub:E212457B-4FB0-48B9-BF64-01826BB2ADA4 *Anomoedus caddoi* LSID: urn:lsid:zoobank.org:act:A2304120-E076-4C6B-A0D3-2D7ED855E68A *Sciroseps* LSID: urn:lsid:zoobank.org:act:D29AE834-7273-453F-AF63-A6C448562C72 *Sciroseps pawhuskai* LSID: urn:lsid:zoobank.org:act:DA0193D8-7270-4BC7-B989-C7E1E6A81B1F.
